# Diastolic shock index and clinical outcomes in patients with septic shock

**DOI:** 10.1186/s13613-020-00658-8

**Published:** 2020-04-16

**Authors:** Gustavo A. Ospina-Tascón, Jean-Louis Teboul, Glenn Hernandez, Ingrid Alvarez, Alvaro I. Sánchez-Ortiz, Luis E. Calderón-Tapia, Ramiro Manzano-Nunez, Edgardo Quiñones, Humberto J. Madriñan-Navia, Juan E. Ruiz, José L. Aldana, Jan Bakker

**Affiliations:** 1 Department of Intensive Care Medicine, Fundación Valle del Lili - Universidad ICESI, Av. Simón Bolívar Cra. 98, Cali, Colombia; 2 Traslational Medicine in Critical Care and Experimental Surgery Laboratory (TransLab-CCM), Universidad ICESI, Cali, Colombia; 3 Service de Réanimation Médicale, Hôpital Bicêtre, Hôpitaux Universitaires Paris–Sud, Paris, France; 4 Assistance Publique Hôpitaux de Paris, Université Paris–Sud, Paris, France; 5 Departamento de Medicina Intensiva, Pontificia Universidad Católica de Chile, Santiago, Chile; 6 Department of Intensive Care Adults, Erasmus MC University Medical Center, Rotterdam, The Netherlands; 7 Department of Pulmonary and Critical Care, New York University, New York, USA; 8 Division of Pulmonary, Allergy, and Critical Care Medicine, Columbia University Medical Center, New York, USA

**Keywords:** Septic shock, Acute circulatory dysfunction, Diastolic shock index, Clinical outcomes

## Abstract

**Background:**

Loss of vascular tone is a key pathophysiological feature of septic shock. Combination of gradual diastolic hypotension and tachycardia could reflect more serious vasodilatory conditions. We sought to evaluate the relationships between heart rate (HR) to diastolic arterial pressure (DAP) ratios and clinical outcomes during early phases of septic shock.

**Methods:**

Diastolic shock index (DSI) was defined as the ratio between HR and DAP. DSI calculated just before starting vasopressors (Pre-VPs/DSI) in a preliminary cohort of 337 patients with septic shock (January 2015 to February 2017) and at vasopressor start (VPs/DSI) in 424 patients with septic shock included in a recent randomized controlled trial (ANDROMEDA-SHOCK; March 2017 to April 2018) was partitioned into five quantiles to estimate the relative risks (RR) of death with respect to the mean risk of each population (assumed to be 1). Matched HR and DAP subsamples were created to evaluate the effect of the individual components of the DSI on RRs. In addition, time-course of DSI and interaction between DSI and vasopressor dose (DSI*NE.dose) were compared between survivors and non-survivors from both populations, while ROC curves were used to identify variables predicting mortality. Finally, as exploratory observation, effect of early start of vasopressors was evaluated at each Pre-VPs/DSI quintile from the preliminary cohort.

**Results:**

Risk of death progressively increased at gradual increments of Pre-VPs/DSI or VPs/DSI (One-way ANOVA, *p* < 0.001). Progressive DAP decrease or HR increase was associated with higher mortality risks only when DSI concomitantly increased. Areas under the ROC curve for Pre-VPs/DSI, SOFA and initial lactate were similar, while mean arterial pressure and systolic shock index showed poor performances to predict mortality. Time-course of DSI and DSI*NE.dose was significantly higher in non-survivors from both populations (repeated-measures ANOVA, *p* < 0.001). Very early start of vasopressors exhibited an apparent benefit at higher Pre-VPs/DSI quintile.

**Conclusions:**

DSI at pre-vasopressor and vasopressor start points might represent a very early identifier of patients at high risk of death. Isolated DAP or HR values do not clearly identify such risk. Usefulness of DSI to trigger or to direct therapeutic interventions in early resuscitation of septic shock need to be addressed in future studies.

## Background

Definition of shock incorporates the presence of low arterial pressure in association with abnormalities in tissue perfusion leading to abnormal oxygen metabolism by the cells [1]. Because the intimate relationship between blood pressure and flow, operational definitions of shock include the fall of mean (MAP) and/or systolic arterial pressure (SAP) [1, 2]. Nevertheless, alterations of pulse wave could grossly mirror, in some extend, the underlying mechanisms of acute circulatory failure implied in shock. For example, SAP results particularly important to define cardiogenic shock [2], hemorrhagic [3] or any type of shock with a hypovolemic component, since at very early stage of these conditions, SAP and pulse pressure (PP) fall while diastolic arterial pressure tends to be sustained. However, hypotension observed during septic shock results from a complex interaction between vasodilation, relative and absolute hypovolemia, myocardial dysfunction, and altered blood flow distribution [4]. In particular, vasodilation resulting from the failure of the vascular smooth muscle to constrict is one of the leading mechanisms associated with hypotension and tissue hypoperfusion in septic shock [5]. In these cases, diastolic arterial pressure (DAP) would better reflect vasodilation than SAP or MAP.

In healthy people, DAP is mainly determined by vascular tone and it remains nearly constant from the ascending aorta to the peripheral vessels [6]. Thus, detection of low DAP at peripheral vessels should reflect systemic vasodilation as long as aortic valve is competent. However, in general, DAP is not considered for definition of septic shock, and with few exceptions, its relationship with clinical outcomes has not been widely described [7]. Important studies in patients with septic shock define hypotension in terms of MAP and SAP values [8–10] assuming the pivotal role of MAP [11] or SAP, on organ perfusion [12–14], in addition to the prognostic value of sustained low MAP values [15]. Nevertheless, evaluation of the loss of vascular tone through the severity of diastolic hypotension could have profound implications on therapeutic decisions since there are not robust clues to rapidly predict when hypotension will be sustainably corrected with fluid loading. Thus, rapid assessment of severity of vasodilation could influence therapeutic decisions such as the early introduction of vasoactive agents [16], which theoretically would avoid unnecessary fluid administration while promptly restoring tissue perfusion.

Remarkably, DAP should not be evaluated separately from heart rate. Acute reductions in arterial pressure are compensated by increased sympathetic activity, although sometimes such compensation becomes maladaptive. This was the original rationale to indexing SAP by heart rate (HR) during hemorrhagic shock and acute critical illness [17, 18], or indexing MAP by HR to detect myocardial hypoperfusion [19]. Likewise, as DAP depends on vascular tone and the duration of the cardiac cycle [20], a combination of DAP and HR could reflect the severity of circulatory dysfunction during vasodilatory conditions. Thus, we evaluated the relationships between very early HR:DAP ratios (i.e., the diastolic shock index, or DSI, calculated just before or at the start of vasopressor support) and clinical outcomes in patients with septic shock, hypothesizing that very early DSI values could promptly identify patients at high risk of unfavorable outcomes, while persistence of high DSI during the first hours of resuscitation could reflect more severe cardiovascular dysfunction.

## Materials and methods

### Study population

A total of 761 patients were analyzed: a preliminary cohort of 337 patients with sepsis requiring vasopressor support (January 2015 to February 2017) from one mixed-ICU in a university hospital in Colombia (Fundación Valle del Lili, Cali, Colombia) and 424 patients with septic shock included in a recent randomized controlled study (March 2017 to April 2018) conducted in 28 hospitals in 5 countries (Argentina, Chile, Colombia, Ecuador, Uruguay), the ANDROMEDA-SHOCK trial [21]. The respective ethical and research committee involving human beings approved the use of the data obtained in both the initial cohort (Protocol number 1238, IRB/EC approval number 099-2018, Fundación Valle del Lili, Cali, Colombia) and the randomized controlled trial [21].

Septic shock was defined in the ANDROMEDA-SHOCK population according to the *Third International Consensus Definitions for Sepsis and Septic Shock* (Sepsis 3.0), which states septic shock as the combination of suspected infection accompanying life-threatening organ dysfunction, requirement of vasopressor therapy to elevate MAP ≥ 65 mmHg and lactate > 2 mmol/L despite adequate fluid resuscitation [22]. Meanwhile, patients from the preliminary cohort were included under the diagnostic criteria for septic shock stated in the Surviving Sepsis Campaign: International Guidelines for Management of Severe Sepsis and Septic Shock: 2012 [23], based on the previous 2001 SCCM/ESICM/ACCP/ATS/SIS International Sepsis Definitions Conference [24], valid during the period in which the database was constructed.

Exclusion criteria for preliminary cohort covered patients < 18-year old, pregnant women, patients with liver failure (protrombin time > 15 s or international normalized ratio ≥ 1.5 and any hepatic encephalopathy), advanced liver cirrhosis (Child–Pugh C), acute/chronic atrial fibrillation, presence of ventricular arrhythmia, use of definitive/transitory pacemaker and those with do-not-resuscitate orders. Meanwhile, exclusion criteria for the ANDROMEDA-SHOCK population are detailed elsewhere [21].

### Study design

DSI was calculated as the quotient between HR and DAP registered just before the start of vasopressor therapy (Pre-VPs/DSI) in the preliminary cohort and at the randomization point in the ANDROMEDA-SHOCK population (< 4 h of septic shock diagnosis according to inclusion criteria), i.e., VPs/DSI [21]. Then, DSI was subsequently calculated 2, 4, and 8 h after the introduction of vasopressor support in both populations. Time elapsed from the first hypotension episode and the first fluid load with resuscitative intention was registered in the preliminary cohort, while time elapsed from the diagnosis of septic shock up to randomization was recorded for the ANDROMEDA-SHOCK population. Most of the initial measurements (i.e., pre-vasopressor and at the start of vasopressor) were obtained by non-invasive techniques using an oscillometric brachial cuff, typically in those patients admitted from the emergency room and general wards. However, invasive pressures were registered later on, when an indwelling intra-arterial catheter was placed. The volume of resuscitation fluids was registered at Pre-VPs point, and then, 2, 4 and 8 h after in the preliminary cohort, and at the VPs/DSI point, and 8 h after in the ANDROMEDA-SHOCK population. Meanwhile, net fluid balance was recorded at 8 and 24 h after the start of vasopressors in both populations. The HR-to-SAP ratio [18, 25] was also calculated at same time points. Multiple organ dysfunction was assessed using the Sequential Organ Failure Assessment Score (SOFA) [26], while ventilator-free days and requirement of acute renal replacement therapy were also registered.

Finally, as a simple exploratory observation, the effect of timing to start vasopressor support was evaluated in the preliminary cohort. A very early start of vasopressor was defined as the one started within the first hour of receiving the first fluid load with resuscitative intention such as it was recently reported [27].

### General management

Patients from the preliminary cohort followed an early quantitative resuscitation protocol adapted from the Surviving Sepsis Campaign [23, 28], aimed in general to target (a) MAP ≥ 65 mmHg; (b) urine output > 0.5 mL/kg/h; (c) ScvO_2_ ≥ 70%, when available; (d) normalization of lactate levels or decreasing of 20% every-2 h in lactate levels. A complete description of the resuscitation protocol and general management in such cohort is described elsewhere [29]. Meanwhile, patients collected from ANDROMEDA-SHOCK trial were randomly allocated to peripheral perfusion-targeted resuscitation or lactate level-targeted resuscitation following a protocol described in detail elsewhere [30].

### Statistical analysis

First, DSI values, calculated just before the start of vasopressors (Pre-VPs/DSI) in the preliminary cohort or at the randomization point (VPs/DSI) in the ANDROMEDA-SHOCK population, were partitioned into five quantiles to estimate the relative risks (RR) of death in relation to the mean risk of their respective population (assumed to be 1). The mean risk and 95% confidence intervals at each DSI quintile were calculated after adjustment for the covariables: age, SOFA score day-1, APACHE II, initial arterial lactate, and volume of resuscitation fluids received before start of vasopressors and from vasopressor start up to 8 h after. Then, new partitions were performed aiming to evaluate the effect of individual components of Pre-VPs/DSI or VPs/DSI (i.e., DAP and HR) on the relative risk of death, as follows: (a) into quintiles of progressively higher DAP; (b) into quintiles of progressively higher HR; (c) re-stratifying each original quintile of DAP into 5 sub-clusters of DSI to extract patients with similar DSI values and therefore, simultaneous increasing of HR and DAP.

Second, repeated-measures ANOVA were used to evaluate differences in the time-course of DSI, mean arterial pressure, DAP, HR, pulse pressure, and vasopressor doses between survivors and non-survivors at day-90 in both preliminary and ANDROMEDA-SHOCK populations. Similarly, the time-course of the product of DSI and dose of vasopressor (DSI*NE.dose) was compared between survivors and non-survivors at day-90.

Third, receiver operating characteristic (ROC) curves were used to identify the performance of variables at pre-VP point (for preliminary cohort) or at randomization point (for ANDROMEDA-SHOCK), and 8 h after, to predict mortality at day-28 and 90. Such variables were Pre-VPs/DSI (or VPs/DSI, in the case of patients from ANDROMEDA-SHOCK), lactate, mean arterial pressure, SOFA score, APACHE II, and systolic shock index (HR:SAP ratio). In addition, the interaction or product of DSI by the dose of vasopressor (DSI*NE.dose) was also included at points where the patients were under vasopressor support.

Fourth, the effect of very early start of vasopressors on mortality at day-90 in each quintile of Pre-VPs/DSI from the preliminary cohort was evaluated using a Chi square test and additionally, logistic regression models adjusted by SOFA score and initial lactate at each Pre-VPs/DSI quintile. A Hosmer and Lemeshow test was used to assess the goodness of fit in each model.

## Results

A total of 761 patients with septic shock were analyzed: 337 patients from a preliminary cohort (Additional file 1: Figure S1a) and 424 from the randomized controlled trial ANDROMEDA-SHOCK (Additional file 1: Figure S1b). A STROBE statement checklist for observational studies is provided in SDC Additional file 1: Table S1. Lengths of ICU and hospital stay were 9 (4–16) and 14 (6–29) days, respectively, in the preliminary cohort, while these were 6 (3–12) and 13 (6–26) days in the ANDROMEDA-SHOCK. Overall mortality at days-28 and 90 were 38.3% and 43.0% in the preliminary cohort, and 39.2% and 43.9% in the ANDROMEDA-SHOCK. General characteristics of both preliminary cohort and ANDROMEDA-SHOCK are presented in the Table 1.

**Table 1:** General characteristics

	Preliminary Cohort (*n* = 337)	Andromeda-Shock (*n* = 424)
General characteristics		
Age, years	64 (51 to 74)	66 (52 to 76)
Male sex, *n* (%)	188 (55.8)	226 (53.3)
Weight, Kg	68 (59 to 76)	70 (59 to 80)
APACHE II	16 (13 to 22)	21 (17 to 28)
SOFA day-1	9 (7 to 12)	10 (7 to 12)
Charlson Comorbidity Index	4 (2 to 5)	3 (1 to 5)
Chronic hypertension, n (%)	73 (39.2)	176 (41.5)
Source of Infection		
Lung	120 (35.6)	128 (30.2)
Genitourinary	60 (17.8)	87 (20.5)
Abdominal	110 (32.6)	149 (35.1)
Other	47 (13.9)	60 (14.2)
Delay time antibiotics, hours	2 (− 2 to 5)	2 (1 to 2)
Time from first fluid resuscitation load up to vasopressor start, hours	2 (0 to 3)	–
Time from hypotension up to vasopressor start, hours	3 (1 to 4)	–
Time from septic shock diagnosis up to randomization, min	–	81 (0 to 180)
At vasopressor start		
SAP	92 (83 to 106)	100 (85 to 113)
DAP	45 (40 to 51)	52 (45 to 60)
MAP	63 (56 to 69)	66 (60 to 76)
HR	104 (87 to 121)	103 (87 to 120)
PP	46 (35 to 59)	45 (35 to 58)
DSI	2.28 (1.83 to 2.74)	1.97 (1.58 to 2.48)
SvO_2_, %, *n*	71.7 (63.8 to 78.2), 196	73.0 (65.0 to 79.0), 401
Pv-aCO_2_, mmHg, *n*	5.0 (3.7 to 7.0), 195	7.0 (5.0 to 10.0), 398
CVP at VPs, mmHg, n	7 (4 to 12), 69	9 (6 to 13), 393
Lactate (initial), mmol/L, *n*	2.7 (1.6 to 4.9), 337	3.5 (2.7 to 5.4), 424
Fluids/VP/RRT		
Volume of resuscitation fluids up to start of VP, mL	1200 (400 to 2000)	2000 (1200 to 2800)
Volume of resuscitation fluids up to start of VP, mL/kg	16.3 (5.7 to 30.0)	27.8 (18.8 to 41.7)
Volume of resuscitation fluids up to 8 h, mL	1050 (1000 to 2500)	1000 (0 to 2000)
Net fluid balance		
At 24 h	2700 (1200 to 4500)	1940 (900 to to 3350)
Norepinephrine max. dose, µg/kg/min	0.26 (0.13 to 0.48)	0.26 (0.11 to 0.45)
Acute RRT	94 (27.9)	72 (17.0)
Clinical outcomes		
ICU LOS	9 (4 to 16)	6 (3 to 12)
Hospital LOS	14 (6 to 29)	13 (6 to 26)
Mechanical ventilation-free days	20 (0 to 27)	16 (0 to 26)
RRT-free days	28 (6 to 28)	28 (2 to 28)
Mortality 28-day, *n* (%)	129 (38.3)	166 (39.2)
Mortality 90-day, *n* (%)	145 (43.0)	186 (43.9)

*Including only patients receiving renal replacement therapy at least for one session

*APACHE II* Acute physiology and chronic health evaluation, *SOFA* sequential organ failure assessment, *VP* vasopressor, *SAP* systolic arterial pressure, *DAP* diastolic arterial pressure, *MAP* mean arterial pressure, *HR* heart rate, *PP* pulse pressure, *DSI* diastolic shock index (HR:DAP ratio), *SvO*_*2*_ oxygen venous saturation, *Pv-aCO*_*2*_ venous-to-arterial carbon dioxide difference, *CVP* central venous pressure, *RRT* renal replacement therapy, *ICU LOS* Intensive Care Unit length of stay, *Hospital LOS* hospital length of stay

**Fig. 1:**
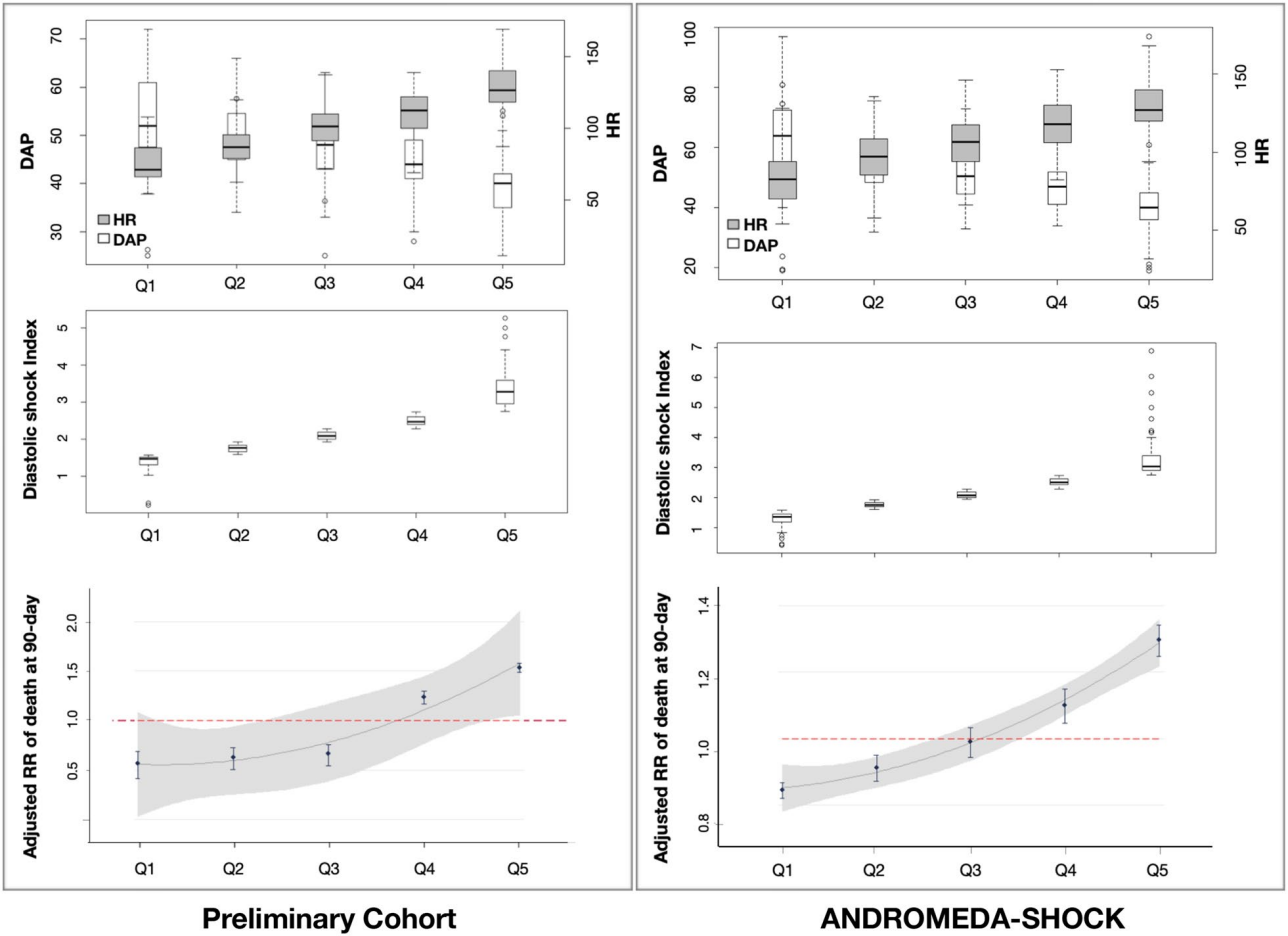
Relative risk of death at day-90 according to pre-vasopressor diastolic shock index (Pre-VPs/DSI) or vasopressor start (VPs/DSI) partitions in the preliminary and ANDROMEDA SHOCK populations. Diastolic shock index values obtained from just before the start of vasopressor (in preliminary cohort) and at the start vasopressor support (in ANDROMEDA-SHOCK) were partitioned into 5 quantiles (Q1 to Q5). Distribution of heart rate (HR) and diastolic pressure (DAP) (top) and their respective diastolic shock index distribution (middle) are presented through the quantile distribution. Boxplots (top and middle) delineate the interquartile range, the median is shown as a line in the middle of the box, and tails represent the 95% range. Coefficients derived from a logistical regression were used to calculate the cut-off value of the diastolic shock index (DSI) detecting the mean risk of mortality of the entire population at 28 days. This point was used as the reference to calculate the adjusted relative risks, in such a way that a relative risk of 1 represents the mean risk of the respective population (bottom). The mean risk and 95% confidence interval (error bars at the bottom) for each percentile were calculated after multivariate adjustment (Cox proportional-hazards model) for the covariables: age, gender, SOFA score day-1, initial arterial lactate and pH, and resuscitation fluids from VP to 8H. The gray zone represents the 95% confidence interval for the Cox regression (continuous line) across the complete population, assuming the diastolic shock index as a continuous variable. Note that adjusted relative risk of death increases as diastolic shock index also does through the quintile distribution

**Fig. 2:**
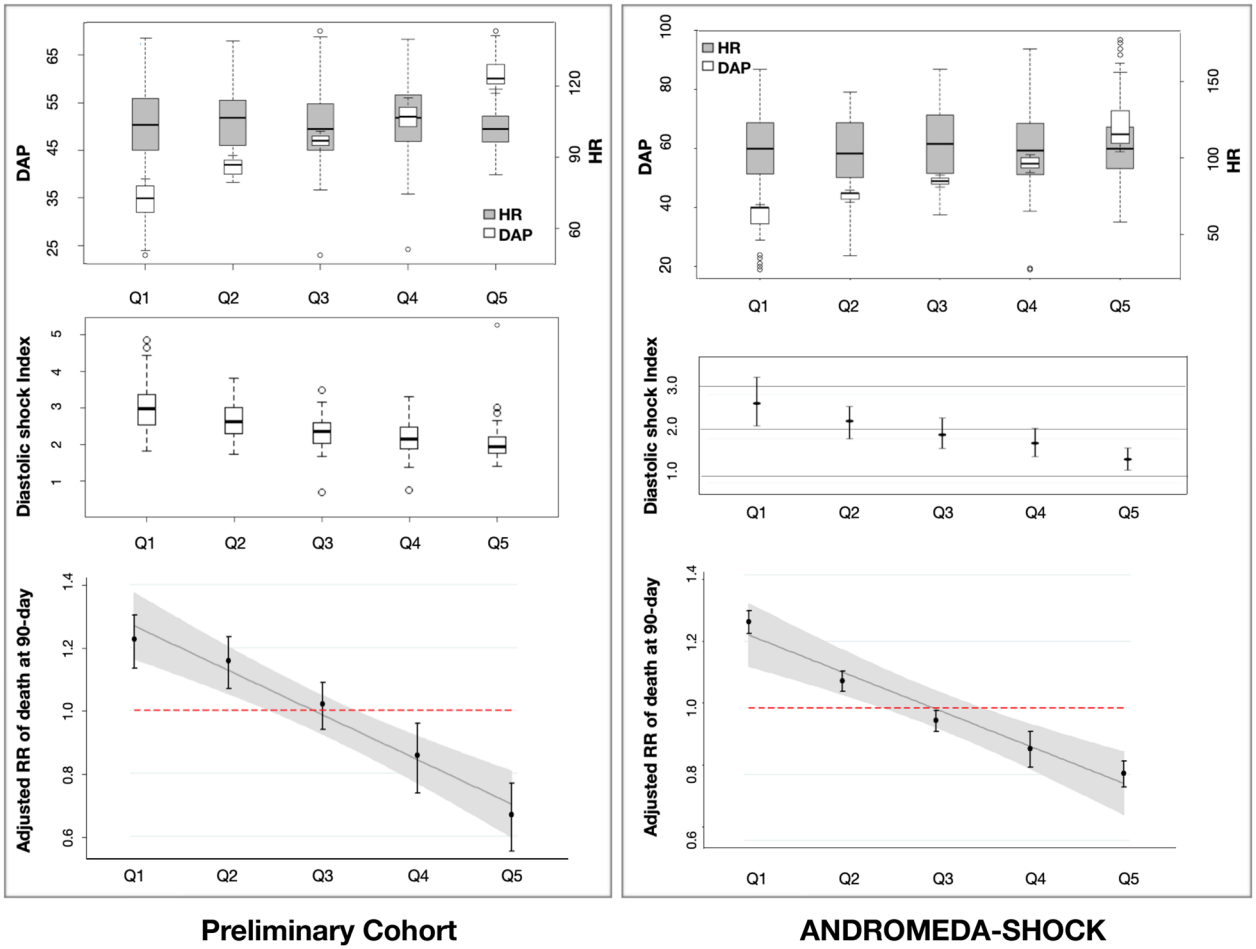
Relative risk of death at day-90 according to diastolic arterial pressure (DAP) partition in the preliminary and ANDROMEDA SHOCK populations. Diastolic arterial pressure (DAP) values from just before the start of vasopressor support were partitioned into 5 quantiles (Q1 to Q5). Distribution of heart rate (HR) and diastolic pressure (DAP) (top) displays a progressive increasing of DAP values through the quantile partitioning with their corresponding HR values, which remains similar from Q1 to Q5. The respective diastolic shock index distribution (middle) is presented through the quantile distribution. The boxes (top) delineate the interquartile range, the median is shown as a line in the middle of the box, and tails represent the 95% range. Boxplots/error bars (middle) represent medians and 95% confidence intervals of the diastolic shock index (DSI) at each quantile. Relative risks’ distributions (bottom) were calculated as described in Fig. 1. Note that adjusted relative risk of death decreases as DAP increases and subsequently DSI decreases, for similar HR values

**Fig. 3:**
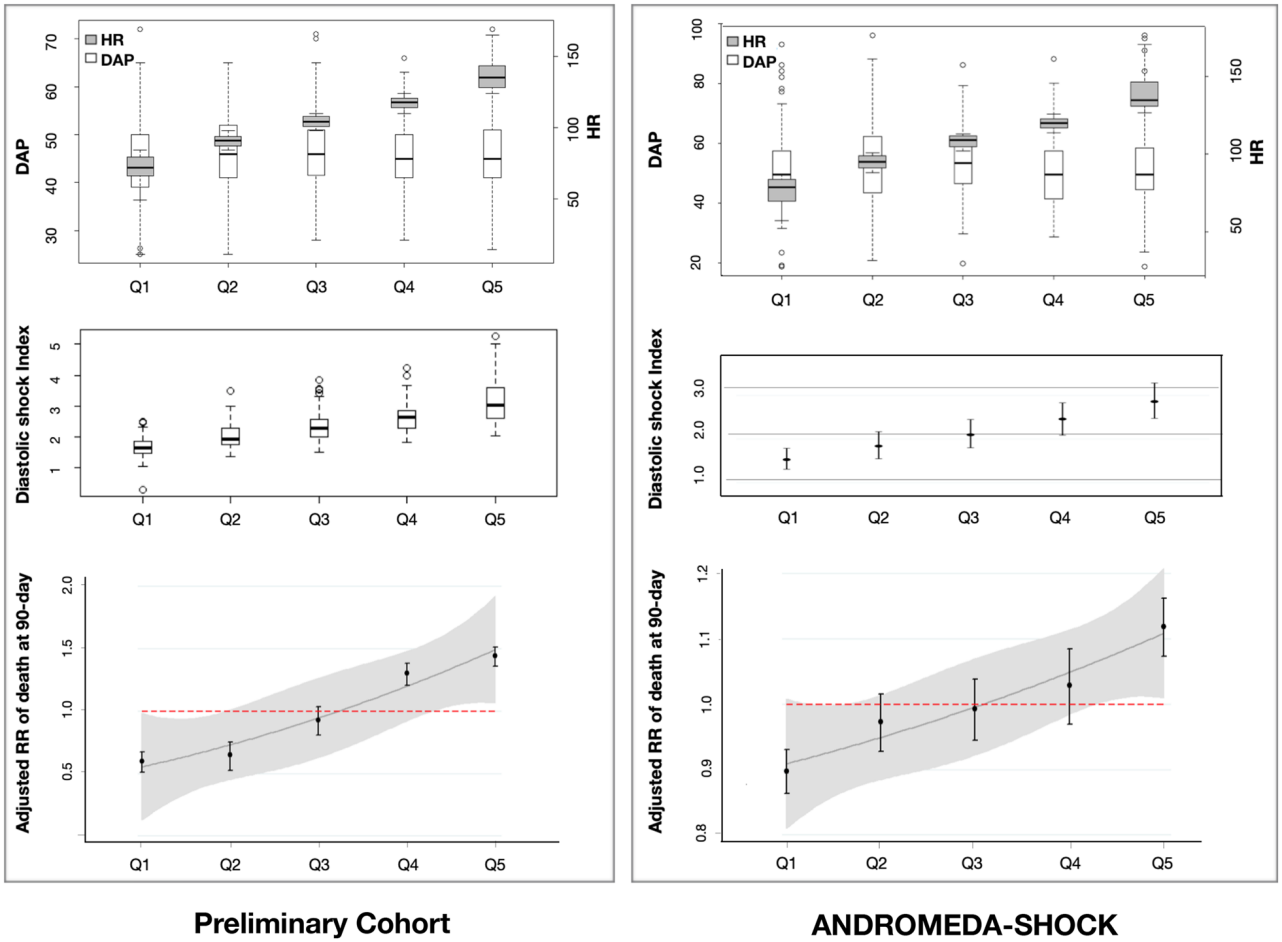
Relative risk of death at day-28 according to heart rate (HR) partition the preliminary and ANDROMEDA SHOCK populations. Heart rate (HR) values from just before the start of vasopressor support were partitioned into 5 quantiles (Q1 to Q5). Distribution of heart rate (HR) and diastolic pressure (DAP) (top) displays a progressive increasing of HR values through the quantile partitioning with their corresponding DAP values, which remains similar from Q1 to Q5. The respective diastolic shock index distribution (middle) is presented through the quantile distribution. The boxes (top) delineate the interquartile range, the median is shown as a line in the middle of the box, and tails represent the 95% range. Boxplots/error bars (middle) represent medians and 95% confidence intervals of the diastolic shock index (DSI) at each quantile. Relative risks’ distributions (bottom) were calculated as described in Fig. 1. Note that adjusted relative risk of death increases as HR and subsequently DSI also increases, for similar DAP values

Progressive increases in Pre-vasopressor DSI (Pre-VPs/DSI) or DSI at vasopressor start (VPs/DSI) were related with gradual increases in the relative risk of death at day-90 in the preliminary and ANDROMEDA-SHOCK populations (Fig. 1). Similar HR values were related with progressively lower risk of death as long as DAP gradually increases, and consequently, DSI values decrease (Fig. 2). Likewise, similar DAP values were related with progressively higher risk of mortality as long as HR gradually increases, and consequently, DSI also did (Fig. 3). Nevertheless, simultaneous increases in HR and DAP with subsequent similar DSI values were related with similar risk of death (Additional file 1: Figure S2). A complete description for DSI, DAP and HR partitioning is presented in the Additional file 1: Tables S2, S3. Meanwhile, a complete description of general demographics, hemodynamics, lactate, renal replacement and mechanical ventilation requirements, resuscitation and cumulative fluids according to the Pre-VPs/DSI and VPs/DSI in the preliminary cohort and ANDROMEDA-SHOCK populations are presented in Additional file 1: Tables S4, S5.

**Fig. 4:**
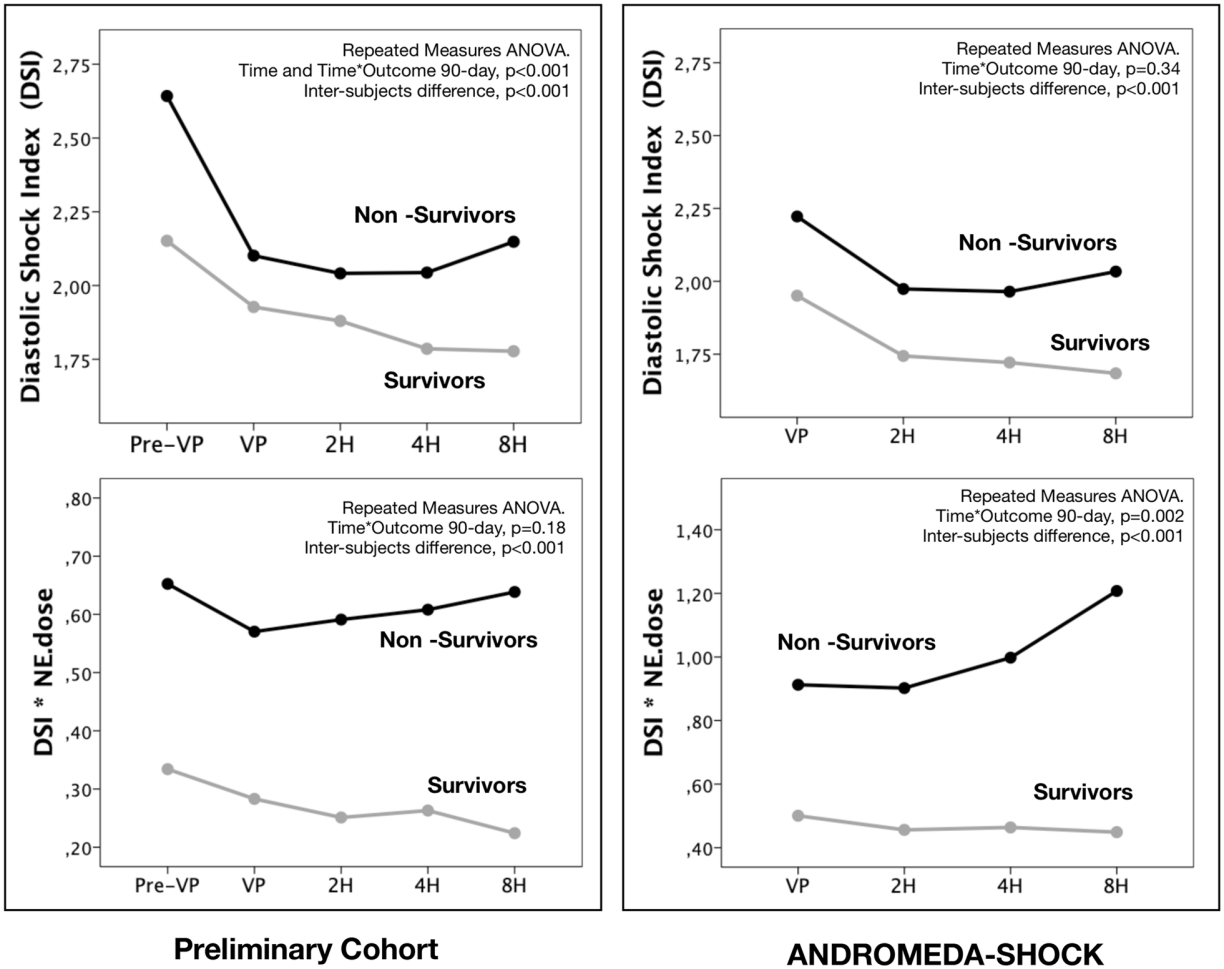
Time-course of diastolic shock index (DSI) and the interaction between DSI and norepinephrine dose for survivors and non-survivors at day-90 in the preliminary cohort and ANDROMEDA-SHOCK. **Left panel, Top.** Time-course of DSI for survivors and non-survivors at day-90 in the preliminary cohort. Repeated-measures ANOVA, Time*Outcome day-90, *p* < 0.001. Inter-subjects difference, *p* < 0.001. **Left panel, Bottom.** Time-course of interaction of DSI and norepinephrine dose for survivors and non-survivors at day-90 in the preliminary cohort. Repeated-measures ANOVA, Time*Outcome day-90, *p* = 0.18. Inter-subjects’ difference, *p* < 0.001. **Right panel, Top.** Time-course of DSI for survivors and non-survivors at day-90 in ANDROMEDA-SHOCK population. Repeated-measures ANOVA, Time*Outcome day-90, *p* = 0.34. Inter-subjects difference, *p* < 0.001. **Right panel, Bottom.** Time-course of interaction of DSI and norepinephrine dose for survivors and non-survivors at day-90 in ANDROMEDA-SHOCK population. Repeated-measures ANOVA, Time*Outcome day-90, *p *= 0.02. Inter-subjects’ difference, *p* < 0.001

There were significant differences in the time-course of DSI between survivors and non-survivors at day-90 in both populations (repeated-measures ANOVA, inter-subjects difference *p* < 0.001) (Fig. 4). Similarly, the product of DSI and dose of norepinephrine (DSI*NE.dose) remained significantly high in non-survivors from both populations (repeated-measures ANOVA, inter-subjects difference *p* < 0.001) (Fig. 4). Time-course of diastolic pressure, heart rate, mean arterial pressure, pulse pressure and systolic shock index for survivors and non-survivors are showed in Additional file 1: Figures S3–S7.

Pre-VPs/DSI from preliminary cohort or VPs/DSI from ANDROMEDA-SHOCK depicted similar performance to predict mortality at day-28 and 90 than other variables such as SOFA score and initial lactate levels (Additional file 1: Figure S8a–S9b). Conversely, mean arterial pressure or isolated diastolic arterial pressure and the systolic shock index showed poor performance for such prediction. DSI and DSI*NE.dose at 8 h showed again similar performances than SOFA score and lactate values, while mean arterial pressure, diastolic arterial pressures and the systolic shock index depicted a poor performance to predict mortality at day-90 (Additional file 1: Figure S8b–S9b).

Very early start of norepinephrine (i.e., norepinephrine started within the first hour of the first fluid load with resuscitative intention) was related with a lower mortality in higher Pre-VPs/DSI (i.e., the Pre-VPs/DSI Quintile-5) (Additional file 1: Tables S6, S7).

## Discussion

Our study retrieves four important findings: (a) progressively higher DSI values calculated just before or at the start of vasopressors are associated with a gradual increase in the risk of death in patients with septic shock; (b) isolated low DAP or high HR values do not clearly identify such risk; (c) non-survivors evolve with persistently high DSI values while requiring higher doses of vasopressors and more resuscitation fluids than survivors; (d) Pre-VPs/DSI and VPs/DSI showed similar performance to SOFA score and initial lactate levels to predict mortality, while mean arterial pressure and systolic shock index did not.

Vasodilation plays a key role in the development of hypotension and tissue hypoperfusion in septic shock [5]. DAP reflects in part the vascular tone when aortic valve is competent. Nevertheless, the duration of the cardiac cycle, the blood volume ejected to the aorta and the arterial compliance also influence DAP [20]. Thus, under isovolemic conditions and constant arterial compliance, shortening diastolic times are associated with higher DAP while a prolonged diastole leads to an opposite effect [20]. Consequently, simultaneous and opposite variations in DAP and HR could suggest more severe cardiovascular dysfunction, with progressively high HR unable to compensate DAP drops as a consequence of gradual decrease in vascular tone. Supporting this, our data suggest that such progressively opposite changes in HR and DAP represent more severe circulatory dysfunction with proportional increases in the relative risk of death.

Persistently low MAP [15, 31] or DAP [7] have been related to worse outcomes in septic shock, while new-onset prolonged sinus tachycardia as a consequence of sympathetic activity has been associated with increased major cardiovascular events, prolonged length of stay [32], and higher mortality rates [33]. Nevertheless, isolated DAP or HR just before or at start of vasopressors was not clearly related with mortality in the preliminary cohort and ANDROMEDA-SHOCK populations. Indeed, at similar HR or DAP values, the risk of death was increased only when DSI concomitantly increased (Figs. 2, 3; Additional file 1: Figure S2). In addition, MAP, SAP and “systolic shock index” (or, HR:SAP ratio) were not related with mortality in both populations (Additional file 1: Figures S8, S9). We hypothesized that although MAP and SAP are used to operatively define septic and another types of shock, initial MAP or SAP does not reflect systemic vasodilation, which is a leading mechanism in septic shock. Although DSI depicted a similar AUC–ROC than SOFA score and initial lactate levels, DSI could add some practical and valuable information about how to intervene the initial hemodynamic condition in sepsis.

Progressively high DSI values calculated just before and at the start of vasopressor support were related with gradual increases in the risk of death. Patients in the higher quintiles of pre-VPs/DSI and VPs/DSI required more renal replacement therapy, depicted higher lactate values and also showed slower lactate decreases over the first 8 h of resuscitation. They also required significantly more resuscitation fluids and higher doses of vasopressors as reflected by the product of DSI and dose of norepinephrine (DSI*NE.dose). We hypothesize that persistently higher DSI values reflect a lack of vascular tone requiring progressively higher doses of vasopressors with an inadequate restoration of tissue perfusion. However, the observational nature of our study hinders the direct effect of variations in vasopressor dose or fluid loading on the DSI since the resuscitation maneuvers in each group were guided targeting MAP but not DAP.

All arterial pressure measurements used for DSI calculations in our study were obviously obtained at the peripheral circulation (i.e., at brachial, femoral or radial sites). Although some disagreement in systolic or mean arterial pressure is observed from the ascending aorta to the peripheral vessels, DAP remains almost constant [6, 34], even during experimental endotoxemic conditions in which a “vascular tone decoupling” from central-to-peripheral circulation can occur [35]. Thus, DAP records obtained at peripheral circulation closely reflect central DAP measurements even during severe inflammatory conditions with increased vasodilation and altered arterial compliance. Although it could be argued that invasive vs. non-invasive measurement methods to measure arterial pressure could influence our results, the bias for DAP measurements is far lower than that observed for SAP [36]. Furthermore, although significant differences in SAP or MAP are observed according to if invasive vs. non-invasive method are used [36], DAP recordings are closer at progressively lower DAP values [35]. Consequently, all these considerations claim against the introduction of considerable errors in DSI calculation when using invasive vs. non-invasive DAP values and also favor the notion of DSI as a global marker of decreased vascular tone since DAP is less influenced by the reflection of pulse waves.

This study may have some important clinical implications. It is unlikely that severe hypotension as a result of severe vasodilation could be reversed by simple fluid administration and instead, unnecessary fluids with subsequent harmful accumulation can occur [37, 38]. Although also considered as “first line intervention”, vasopressors are usually used as a rescue therapy when initial fluid administration fails to correct hypotension or when arterial pressure is judged to be insufficient to ensure an adequate tissue perfusion. Recent experimental and observational data suggest that very early start of vasopressor support could be beneficial [27, 39]. Nevertheless, there are no clear signals indicating when vasopressor support should be started. In this way, very early signals of severe vasodilation should alert on its possible immediate requirement. Thus, DSI should not be interpreted as “another index of death”. Instead, a higher DSI value at presentation of severe cases of sepsis could identify patients who might benefit from some very early interventions capable of modifying the course of septic shock. Our data suggest some beneficial of very early start of vasopressors in patients at the higher pre-VPs DSI. Nevertheless, sample size and the retrospective nature of such observation simply pose a hypothesis to be tested in the future.

Our study has several limitations. First, as previously mentioned, its retrospective nature might limit the conclusions since some confounding factors and potential bias may not have been controlled. Nevertheless, observations from preliminary cohort, corroborated in prospectively collected data from a recent randomized controlled trial, reinforce the strength of DSI as an early identifier of septic patients at high risk of death. Second, we did not include a control group of normal subjects, so recognizing a DSI cutoff to identify abnormality could be misleading. Third, although persistently high DSI values were consistently observed in non-survivors in both the preliminary and ANDROMEDA-SHOCK groups, there are no clues about whether it is possible to intervene DSI course or even whether modifying DSI course might influence clinical outcomes. Nevertheless, this could be an important research question as recent experimental observations suggest that some early therapeutic interventions might modify the time-course of cardiovascular dysfunction in septic shock. Finally, despite the apparent plausibility of DSI at very early stages of septic shock, our observations are limited to a relative small sample of patients. Consequently, the potential utility of DSI in the clinical practice should be additionally explored.

## Conclusion

DSI calculated just before or at the vasopressor start might identify patients with septic shock at high risk of death. Isolated DAP or high HR is not clearly related with such risk. Whether the DSI could be used as a trigger or to direct therapeutic interventions in septic shock or sepsis-related cardiovascular dysfunction deserves future research efforts.

## Supplementary information

**Additional file 1.** Additional tables and figures.

## Data Availability

The datasets generated and/or analyzed during the current study are not publicly available as recommended by the local Ethical and research committee involving human beings (Fundación Valle del Lili, Cali, Colombia). Nevertheless, it could be available from the corresponding author on reasonable request and under prior approval by such committee.

## Abbreviations

HR: Heart rate
DAP: Diastolic arterial pressure
MAP: Mean arterial pressure
SAP: Systolic arterial pressure
DSI: diastolic shock index
SOFA: Sequential Organ Failure Assessment Score
APACHE II: Acute Physiology and Chronic Health Evaluation
