# Supplementary material for: Diastolic shock index and clinical outcomes in patients with septic shock
Source: Ann Intensive Care. 2020 Apr 16;10:41. doi: 10.1186/s13613-020-00658-8 (PMC7160223; doi:10.1186/s13613-020-00658-8)
Supplement: Supplementary file 1 — Additional file 1. Additional tables and figures. [file 13613_2020_658_MOESM1_ESM.docx]

**Diastolic shock index and clinical outcomes in patients with septic shock**

**Supplemental Digital Content (SDC)**

**Address for correspondence:** Dr. Gustavo A. Ospina-Tascón

Department of Intensive Care Medicine

Traslational Medicine in Critical Care and Experimental Surgery Laboratory (TransLab CCM)

Fundación Valle del Lili - Universidad ICESI

Av. Simón Bolívar Cra. 98

Cali. Colombia

Tel (+57).2.331.9090 –

Fax (+57).2.331.9090 ext.4237

Email: [gusospin@gmail.com](mailto:gusospin@gmail.com)

**Figure S1a. Selection of patients – Preliminary cohort**

**
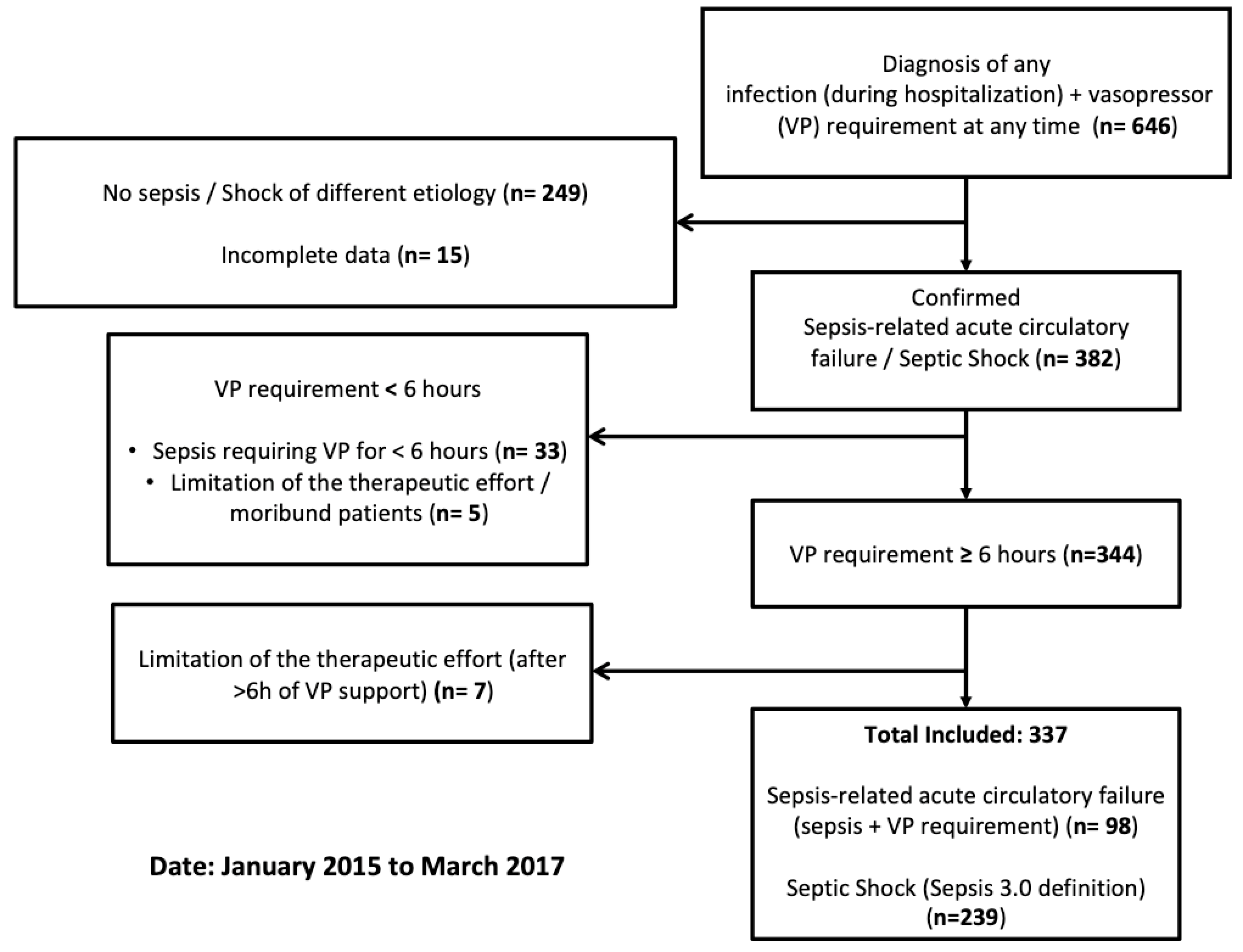
**

**Figure S1b. Selection of patients – Validation group (Andromeda-Shock study)**

**
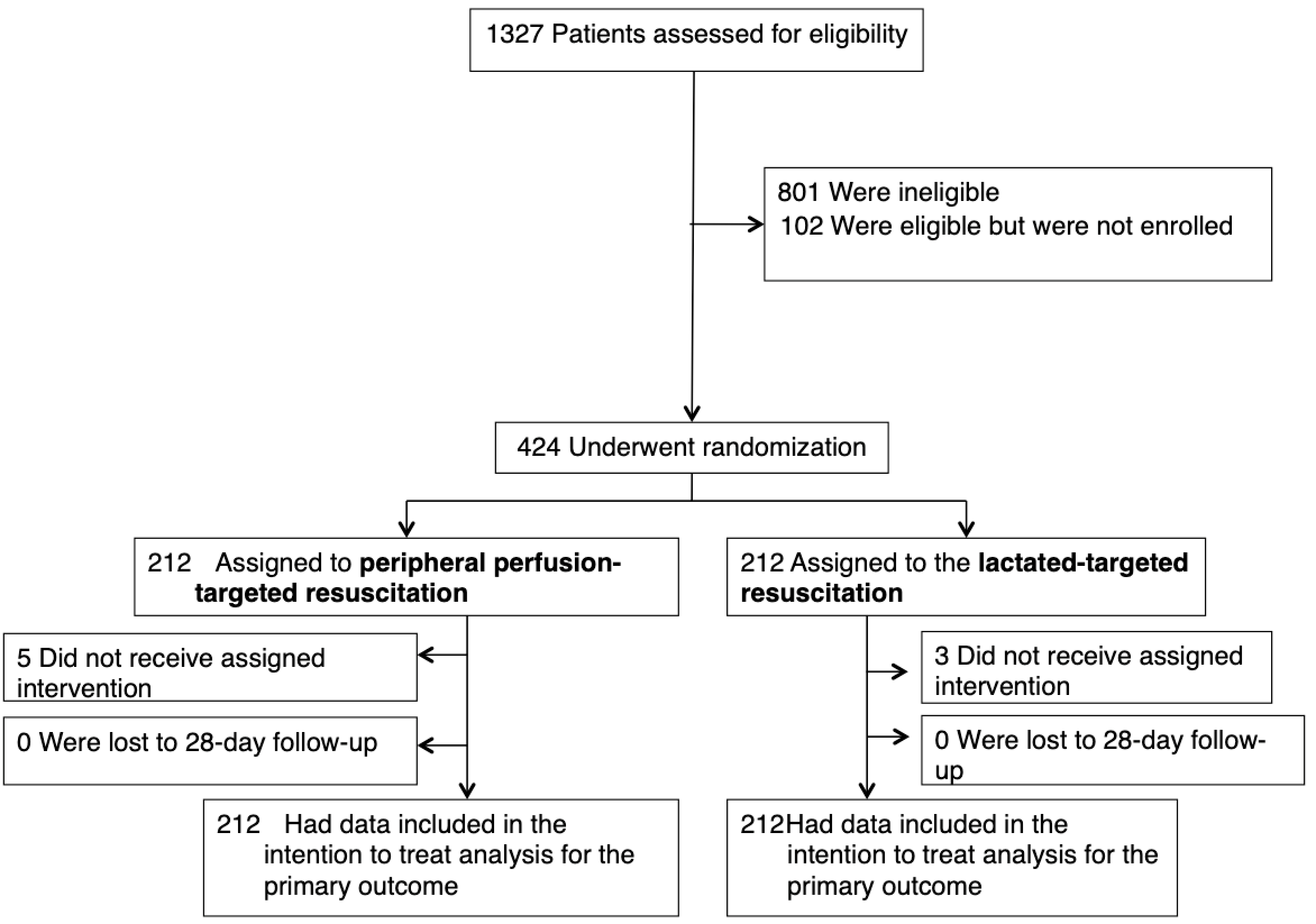
**

**Table S1.** ESTROBE Statement—Checklist for observational studies

|  | Item No | Recommendation | **Action / Fulfillment** |
| --- | --- | --- | --- |
| **Title and abstract** | 1 | (*a*) Indicate the study’s design with a commonly used term in the title or the abstract | Title was not modified |
|  |  | (*b*) Provide in the abstract an informative and balanced summary of what was done and what was found |  |
| Introduction |  |  |  |
| Background/rationale | 2 | Explain the scientific background and rationale for the investigation being reported |  |
| Objectives | 3 | State specific objectives. including any pre-specified hypotheses |  |
| Methods |  |  |  |
| Study design | 4 | Present key elements of study design early in the paper |  |
| Setting | 5 | Describe the setting. locations. and relevant dates. including periods of recruitment. exposure. follow-up. and data collection |  |
| Participants | 6 | (*a*) Give the eligibility criteria. and the sources and methods of selection of participants. Describe methods of follow-up | Flow chart to select participants included |
|  |  | (*b*) For matched studies. give matching criteria and number of exposed and unexposed | Explained in the text |
| Variables | 7 | Clearly define all outcomes. exposures. predictors. potential confounders. and effect modifiers. Give diagnostic criteria. if applicable |  |
| Data sources/ measurement | 8* | For each variable of interest. give sources of data and details of methods of assessment (measurement). Describe comparability of assessment methods if there is more than one group |  |
| Bias | 9 | Describe any efforts to address potential sources of bias |  |
| Study size | 10 | Explain how the study size was arrived at | Sample by convenience |
| Quantitative variables | 11 | Explain how quantitative variables were handled in the analyses. If applicable. describe which groupings were chosen and why | Partitioning by quantiles described in the text |
| Statistical methods | 12 | (*a*) Describe all statistical methods. including those used to control for confounding |  |
|  |  | (*b*) Describe any methods used to examine subgroups and interactions |  |
|  |  | (*c*) Explain how missing data were addressed |  |
|  |  | (*d*) If applicable. explain how loss to follow-up was addressed |  |
|  |  | (*e*) Describe any sensitivity analyses | Do not apply |
| Results |  |  |  |
| Participants | 13* | (a) Report numbers of individuals at each stage of study—eg numbers potentially eligible. examined for eligibility. confirmed eligible. included in the study. completing follow-up. and analysed | Flow chart shown |
|  |  | (b) Give reasons for non-participation at each stage |  |
|  |  | (c) Consider use of a flow diagram | Provided in the ESM |
| Descriptive data | 14* | (a) Give characteristics of study participants (eg demographic. clinical. social) and information on exposures and potential confounders |  |
|  |  | (b) Indicate number of participants with missing data for each variable of interest |  |
|  |  | (c) Summarise follow-up time (eg. average and total amount) |  |
| Outcome data | 15* | Report numbers of outcome events or summary measures over time | Information about ICU, In-hospital and days-28 -90 mortality is provided. Length of stay (ICU and Hospital) |
| Main results | 16 | (*a*) Give unadjusted estimates and. if applicable. confounder-adjusted estimates and their precision (eg. 95% confidence interval). Make clear which confounders were adjusted for and why they were included |  |
|  |  | (*b*) Report category boundaries when continuous variables were categorized | Information about categorization is provided |
|  |  | (*c*) If relevant. consider translating estimates of relative risk into absolute risk for a meaningful time period | Relative risk according to the mortality of each population studied |
| Other analyses | 17 | Report other analyses done—eg analyses of subgroups and interactions. and sensitivity analyses |  |
| Discussion |  |  |  |
| Key results | 18 | Summarize key results with reference to study objectives |  |
| Limitations | 19 | Discuss limitations of the study. taking into account sources of potential bias or imprecision. Discuss both direction and magnitude of any potential bias | Discussed in main text |
| Interpretation | 20 | Give a cautious overall interpretation of results considering objectives. limitations. multiplicity of analyses. results from similar studies. and other relevant evidence |  |
| Generalizability | 21 | Discuss the generalizability (external validity) of the study results |  |
| Other information |  |  |  |
| Funding | 22 | Give the source of funding and the role of the funders for the present study and. if applicable. for the original study on which the present article is based |  |

**Figure S2.** **Relative risk of death at day-90 in clusters of patients with simultaneous and progressive increase in heart rate (HR) and diastolic arterial pressure (DAP).**

**
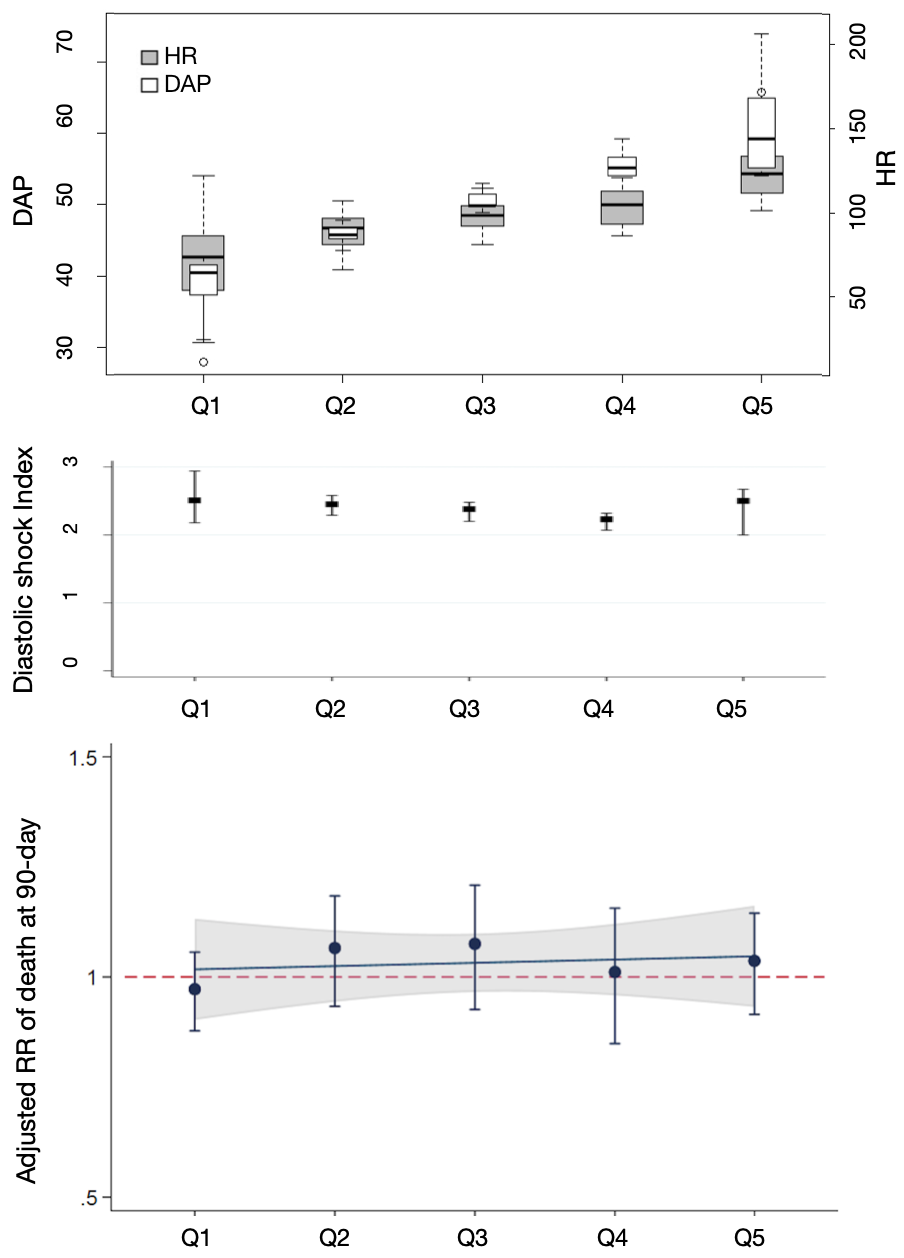
**

The combined population (preliminary cohort and ANDROMEDA-SHOCK) was firstly stratified into quintiles of diastolic arterial pressure (DAP) at the vasopressor start. Then, each quantile of DAP was sub partitioned in 5 clusters of diastolic shock index values (VPs/DSI). Thereafter, a match for clusters with similar DSI values through the Q1 to Q5 DAP distribution was performed. Consequently, clusters with simultaneous and progressive increases in heart rate (HR) and DAP were rearranged (top), with similar diastolic shock index distributions (middle). The boxes (top) delineate the interquartile range, the median is shown as a line in the middle of the box, and tails represent the 95% range. Error bars (middle) represent medians and 95% confidence intervals of the diastolic shock index (DSI) at each quantile.

Coefficients derived from a logistical regression were used to calculate the cut-off value of the diastolic shock index (DSI) detecting the mean risk of mortality of the entire population at 28-day. This point was used as reference to calculate the adjusted relative risks, in such a way that a relative risk of 1 represents the mean risk of the respective population (bottom). The mean risk and 95% confidence interval (error bars at the bottom) for each percentile were calculated after multivariate adjustment (Cox proportional-hazards model) for the covariables: age, gender, SOFA score day-1, initial arterial lactate and pH, and resuscitation fluids from VP to 8H. The gray zone represents the 95% confidence interval for the Cox regression (continuous line) across the complete population, assuming the diastolic shock index as a continuous variable.

Note that adjusted relative risk of death increases as diastolic shock index also does through the quintile distribution.

**Table S2. Distributions of DSI, DAP and HR partitions by quintiles. Preliminary cohort**

|  | **Q1** | **Q2** | **Q3** | **Q4** | **Q5** | **Kruskal Wallis,**  **p** |
| --- | --- | --- | --- | --- | --- | --- |
| **Partitioned by Diastolic Shock Index** | | | | | | |
| Diastolic Shock Index | 1.53 (1.46 – 1.63) | 1.92 (1.83 – 2.00) | 2.28 (2.19 – 2.37) | 2.63 (2.51 – 2.74) | 3.37 (3.13 – 3.65) | <0.001 |
| DAP | 52 (47 - 61) | 49 (45 - 53) | 46 (42 - 50) | 43 (41 - 46) | 38 (34 - 42) | <0.001 |
| HR | 78 (67 - 89) | 91 (85 - 102) | 105 (94 - 114) | 115 (104 - 126) | 128 (119 - 141) | <0.001 |
| **Partitioned by DAP** | | | | | | |
| Diastolic Shock Index | 3.02 (2.44 – 3.47) | 2.58 (2.18 – 3.03) | 2.28 (1.87 – 2.53) | 2.02 (1.69 – 2.43) | 1.74 (1.52 – 2.06) | <0.001 |
| DAP | 35 (32 – 37) | 42 (41 – 42) | 46 (45 – 46) | 50 (49 – 51) | 58 (55 – 62) | <0.001 |
| HR | 101 (85 – 119) | 109 (88 – 124) | 103 (87 – 114) | 101 (84 – 122) | 103 (92 – 122) | 0.468 |
| **Partitioned by HR** | | | | | | |
| Diastolic Shock Index | 1.62 (1.46 – 1.81) | 1.95 (1.73 – 2.26) | 2.28 (2.00 – 2.57) | 2.62 (2.30 – 2.90) | 3.05 (2.61 – 3.59) | <0.001 |
| DAP | 45 (39 – 49) | 46 (41 – 52) | 46 (41 – 52) | 45 (42 – 50) | 45 (41 – 51) | 0.898 |
| HR | 71 (66 – 79) | 90 (87 – 94) | 104 (100 – 106) | 118 (113 – 121) | 136 (129 – 145) | <0.001 |

HR: heart rate; DAP: diastolic arterial pressure

**Table S3. Distributions of DSI, DAP and HR partitions by quintiles. ANDROMEDA-SHOCK**

|  | **Q1** | **Q2** | **Q3** | **Q4** | **Q5** | **Kruskal Wallis,**  **p** |
| --- | --- | --- | --- | --- | --- | --- |
| **Partitioned by Diastolic Shock Index** | | | | | | |
| Diastolic Shock Index | 1.29 (1.10 – 1.40) | 1.67 (1.57 – 1.74) | 1.97 (1.7 – 2.03) | 2.35 (2.22 – 2.48) | 2.98 (2.77 – 3.23) | <0.001 |
| DAP | 65 (56 – 74) | 57 (50 – 62) | 53 (47 – 58) | 48 (42 – 52) | 41 (38 – 47) | <0.001 |
| HR | 81 (67 – 92) | 93 (83 – 106) | 106 (91 – 113) | 112 (99 – 126) | 125 (116 – 137) | <0.001 |
| **Partitioned by DAP** | | | | | | |
| Diastolic Shock Index | 2.65 (2.25 – 3.21) | 2.18 (1.82 – 2.67) | 2.08 (1.75 – 2.34) | 1.81 (1.60 – 2.01) | 1.38 (1.19 – 1.57) | <0.001 |
| DAP | 40 (35 – 40) | 46 (45 – 48) | 52 (50 – 53) | 58 (56 – 60) | 70 (65 – 77) | <0.001 |
| HR | 103 (88 – 120) | 108 (84 – 122) | 107 (90 – 121) | 108 (93 – 118) | 97 (85 – 112) | 0.154 |
| **Partitioned by HR** | | | | | | |
| Diastolic Shock Index | 1.40 (1.18 – 1.71) | 1.71 (1.43 – 2.04) | 2.00 (1.75 – 2.22) | 2.26 (1.93 – 2.67) | 2.67 (2.38 – 3.03) | <0.001 |
| DAP | 50 (45 – 58) | 54 (44 – 63) | 52 (45 – 59) | 52 (43 – 59) | 50 (45 – 59) | 0.883 |
| HR | 75 (66 – 80) | 90 (87 – 94) | 103 (100 – 106) | 115 (101 – 120) | 132 (128 – 144) | <0.001 |

HR: heart rate; DAP: diastolic arterial pressure

**Table S4. General demographics, hemodynamics, lactate, renal replacement / mechanical ventilation requirement and fluids by quintiles of pre-VPs/DSI. Preliminary cohort**

| **Variable** | **Q1** | **Q2** | **Q3** | **Q4** | **Q5** | **P *** |
| --- | --- | --- | --- | --- | --- | --- |
| Age | 63 (51 – 74.0) | 65 (59 – 75) | 64 (55 – 73) | 62 (41 – 72) | 62 (47 – 73) | 0.30 |
| Charlson Scale | 4 (2 – 6) | 4 (3 – 5) | 4 (2 – 5) | 3 (2 – 5) | 3 (2 – 5) | 0.34 |
| APACHE II | 17 (11 – 21) | 16 (13 – 24) | 16 (13 – 21) | 17 (13 – 22) | 16 (13 – 23) | 0.76 |
| SOFA Day 1 | 9 (8 – 12) | 9 (6 – 11) | 9 (7 – 12) | 10 (8 – 12) | 10 (8 – 13) | 0.25 |
| DAP pre-VP, mmHg | 52 (47 - 61) | 49 (45 - 53) | 46 (42 - 50) | 43 (41 - 46) | 38 (34 – 42) | <0.001 |
| HR pre-VP, bpm | 78 (67 - 89) | 91 (85 - 102) | 105 (94 - 114) | 115 (104 - 126) | 128 (119 - 141) | <0.001 |
| MAP pre-VP, mmHg | 67 (61 – 74) | 64 (59 – 71) | 63 (59 – 66) | 59 (55 – 63) | 56 (50 – 64) | <0.001 |
| MAP at the first Fluid resuscitation load, mmHg, n | 62 (56 – 67) | 60 (56 – 66) | 59 (54 – 65) | 59 (54 – 65) | 54 (48 – 59) | 0.12 |
| PP pre-VP, mmHg | 43 (35 – 59) | 44 (35 – 56) | 46 (33 – 65) | 49 (38 – 58) | 48 (38 – 59) | 0.54 |
| Diastolic Shock Index (Pre-VPs/DSI) | 1.53  (1.46 – 1.63) | 1.92  (1.83 – 2.00) | 2.28  (2.19 – 2.37) | 2.63  (2.51 – 2.74) | 3.37  (3.13 – 3.65) | <0.001 |
| pH arterial at start | 7.34  (7.25 – 7.41) | 7.34  (7.26 – 7.42) | 7.32  (7.25 – 7.39) | 7.36  (7.28 – 7.43) | 7.33  (7.22 – 7.41) | 0.18 |
| Lactate at start, mmol/L | 1.9 (1.9 – 4.0) | 2.5 (1.5 – 4.6) | 2.6 (2.2 – 4.5) | 3.0 (2.0 – 4.9) | 4.2 (1.9 – 6.1) | 0.003 |
| Lactate 8-hours, mmol/L | 1.5 (1.0 – 3.4) | 1.9 (1.2 – 3.2) | 1.8 (1.1 – 3.7) | 2.6 (1.4 – 4.1) | 3.4 (1.8 – 6.4) | 0.001 |
| Norepinephrine max. dose, ugr/kg/min | 0.20  (0.12 – 0.43) | 0.24  (0.13 – 0.39) | 0.28  (0.13 – 0.53) | 0.27  (0.14 – 0.44) | 0.45  (0.21 – 0.83) | 0.004 |
| Volume of resuscitation fluids,  Pre-VPs, ml | 800  (230 – 1,500) | 780  (250 – 1,400) | 1,100  (300 – 2,200) | 1,450  (760 – 2,260) | 1,600  (500 – 2,400) | <0.001 |
| Volume of resuscitation fluids,  Pre-VPs, ml/kg | 11.6 (3.4 – 24.1) | 9.9 (3.9 – 24.5) | 16.6 (5.4 – 31.4) | 21.1 (12.5 – 34.5) | 24.6 (5.7 – 37.4) | <0.001 |
| Volume resuscitation fluids, VPs to 8H, ml | 1,650  (1,060 – 2,600) | 1,705  (800 – 2,415) | 1,835  (900 – 3.050) | 2,170  (1,250 – 3,310) | 2,700  (1,300 – 4,000) | 0.005 |
| Net Fluid Balance, at 8H, ml | 2,600  (1,600 – 5,000) | 1,950  (1,000 – 3,400) | 2,970  (1,820 – 6,130) | 2,570  (1,470 – 4,230) | 3,500  (1,480 – 6,140) | 0.011 |
| Net Fluid Balance, at 24H, ml | 4,550  (2,830 – 6,900) | 3,800  (2,130 – 5,160) | 4,750  (3,515 – 7,400) | 5,170  (3,350 – 7,530) | 6,300  (4,000 – 8,800) | <0.001 |
| Mechanical ventilation-free days | 23 (12 – 28) | 22 (13 – 28) | 21 (0 – 28) | 10 (0 – 24) | 1 (0 – 24) | <0.001 |
| Renal replacement therapy requirement, n (%) | 16 (23.5) | 17 (25.4) | 14 (21.2) | 24 (34.8) | 23 (34.3) | 0.26 |
| Renal replacement therapy – free days | 28 (28 – 28) | 27 (28 – 28) | 28 (28 – 28) | 28 (19 – 28) | 28 (6 – 28) | 0.26 |
| Length of ICU stay, days | 9 (6 – 20) | 10 (5 – 16) | 9 (6 – 16) | 8 (4 – 15) | 3 (3 – 15) | 0.04 |
| Length of hospital stay, days | 19 (11 – 33) | 18 (10 – 31) | 15 (7 – 24) | 11 (5 – 33) | 6 (3 – 19) | <0.001 |
|  |  |  |  |  |  |  |

APACHE II: Acute physiology and chronic health evaluation ; SOFA: sequential organ failure assessment score; HR: heart rate; DAP: diastolic arterial pressure; MAP: mean arterial pressure; PP: pulse pressure; VPs: start of vasopressors

**Table S5. General demographics, hemodynamics, lactate, renal replacement / mechanical ventilation requirement and fluids by quintiles of pre-VPs/DSI. ANDROMEDA-SHOCK**

| **Variable** | **Q1** | **Q2** | **Q3** | **Q4** | **Q5** | **p *** |
| --- | --- | --- | --- | --- | --- | --- |
| Age | 69 (56 – 78) | 68 (54 – 77) | 67 (54 – 77) | 64 (48 – 73) | 62 (49 – 73) | 0.16 |
| Charlson Scale | 3 (1 – 5) | 4 (2 – 5) | 3 (1 – 5) | 3 (1 – 5) | 2 (1 – 5) | 0.66 |
| APACHE II | 19 (15 – 24) | 23 (15 – 24) | 20 (16 – 28) | 23 (18 – 29) | 23 (19 – 29) | 0.001 |
| SOFA Day 1 | 9 (7 – 11) | 9 (7 – 11) | 9 (6 – 11) | 10 (8 – 12) | 10 (8 – 13) | 0.009 |
| DAP at VPs, mmHg | 65 (56 – 74) | 57 (50 – 62) | 53 (47 – 58) | 48 (42 – 52) | 41 (38 – 47) | <0.001 |
| HR at VPs, bpm | 81 (67 – 92) | 93 (83 – 106) | 106 (91 – 113) | 112 (99 – 126) | 125 (116 – 137) | <0.001 |
| MAP at VPs, mmHg | 81 (71 – 90) | 71 (65 – 80) | 67 (63 – 74) | 63 (57 – 66) | 56 (52 – 62) | <0.001 |
| PP at VP, mmHg | 50 (37 – 61) | 45 (37 – 57) | 45 (35 – 57) | 45 (33 – 58) | 40 (32 – 62) | 0.37 |
| Diastolic Shock Index | 1.29  (1.10 – 1.40) | 1.67  (1.57 – 1.74) | 1.97  (1.7 – 2.03) | 2.35  (2.22 – 2.48) | 2.98  (2.77 – 3.23) | <0.001 |
| pH arterial at start | 7.34  (7.25 – 7.41) | 7.34  (7.26 – 7.42) | 7.32  (7.25 – 7.39) | 7.36  (7.28 – 7.43) | 7.33  (7.22 – 7.41) | 0.35 |
| Lactate at start, mmol/L | 3.1 (2.7 – 4.0) | 3.4 (2.7 – 5.5) | 3.5 (2.6 – 5.3) | 4.2 (2.9 – 6.4) | 4.2 (3.2 – 6.2) | <0.001 |
| Lactate 8-hours, mmol/L | 1.9 (1.6 – 2.7) | 2.4 (1.8 – 4.0) | 2.6 (1.8 – 3.9) | 2.4 (1.7 – 5.2) | 3.2 (2.1 – 5.5) | 0.001 |
| Norepinephrine at start, ugr/kg/min | 0.15  (0.09 – 0.22) | 0.20  (0.10 – 0.40) | 0.25  (0.11 – 0.40) | 0.25  (0.12 – 0.46) | 0.25  (0.17 – 0.42) | <0.001 |
| Volume of resuscitation fluids, pre-VPs, ml | 2,000  (1,000 – 2,500) | 2,000  (1,500 – 2,840) | 2,000  (1,275 – 2,500) | 2,000  (1,500 – 3,000) | 2,000  (1,030 – 2,870) | 0.25 |
| Volume of resuscitation fluids, pre-VPs, ml/kg | 25.0  (14.8 – 33.8) | 30.0  (21.4 – 42.3) | 25.0  (18.7 – 36.4) | 30.1  (20.8 – 42.8) | 28.0  (17.3 – 42.9) | 0.15 |
| Volume resuscitation fluids, to 8H, ml | 1,840  (1,040 – 2,920) | 2,190  (1,260 – 3,410) | 2,160  (1,390 – 3,030) | 2,510  (1,700 – 4,050) | 2,620  (1,700 – 3,930) | 0.002 |
| Net Fluid Balance, at 24H, ml | 1,680  (760 – 2,570) | 1,510  (640 – 2,990) | 2,030  (910 – 3,500) | 2,210  (1,150 – 3,890) | 2,100  (995 – 4,250) | 0.14 |
| Mechanical ventilation-free days | 25 (1 – 28) | 12 (0 – 26) | 17 (0 – 27) | 14 (0 – 26) | 2 (0 – 24) | 0.001 |
| Renal replacement therapy requirement, n (%) | 7 (8.4) | 18 (21.4) | 15 (17.9) | 19 (22.9) | 12 (14.5) | 0.004 |
| Length of ICU stay, days | 6 (3 – 11) | 5 (3 – 11) | 6 (3 – 13) | 8 (3 – 12) | 6 (2 – 13) | 0.74 |
| Length of hospital stay, days | 15 (8 – 26) | 11 (5 – 19) | 18 (7 – 31) | 14 (7 – 30) | 10 (3 – 22) | 0.022 |
|  |  |  |  |  |  |  |

APACHE II: Acute physiology and chronic health evaluation ; SOFA: sequential organ failure assessment score; HR: heart rate; DAP: diastolic arterial pressure; MAP: mean arterial pressure; PP: pulse pressure; VPs: start of vasopressors

**Figure S3. Time-course of diastolic arterial pressure (DAP) for survivors and non-survivors at day-90 in the preliminary cohort and ANDROMEDA-SHOCK**


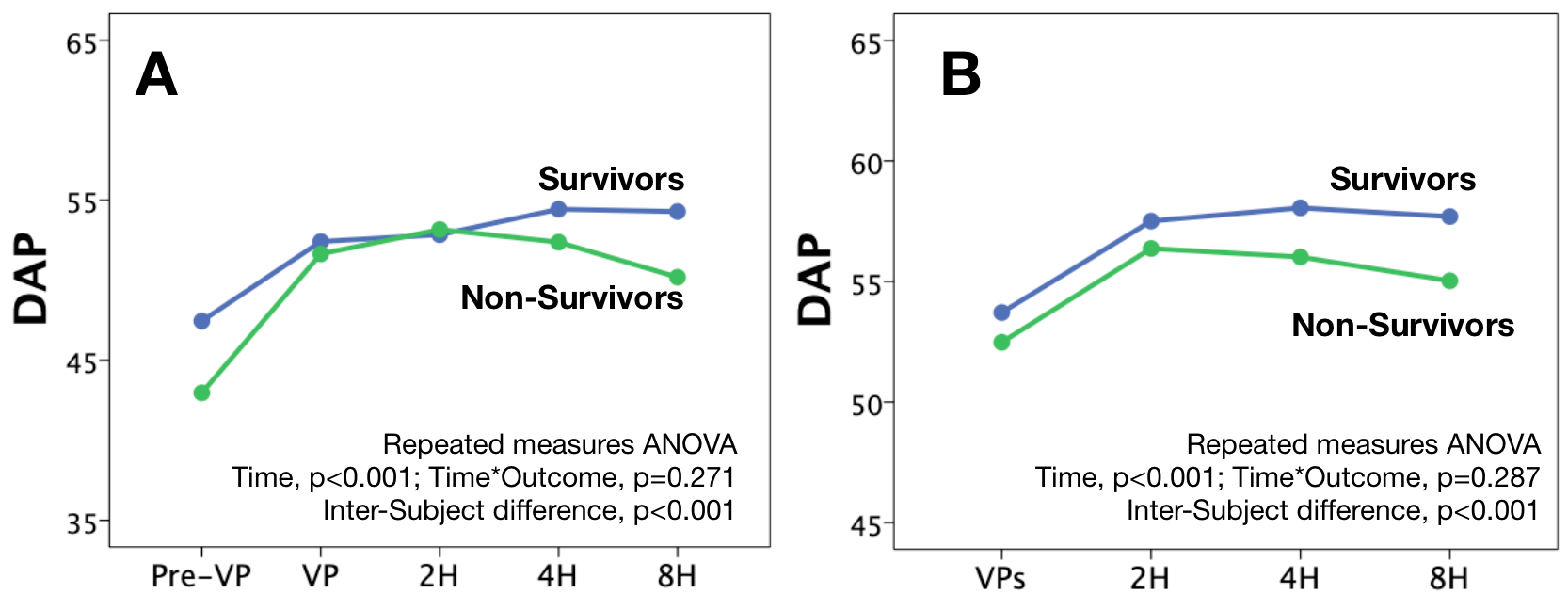


**Panel A.** Repeated Measures ANOVA, preliminary cohort. Inter-group differences (survivors vs. non-survivors at day-90). p<0.001; Time*Outcome. p=0.271

**Panel B.** Repeated Measures ANOVA, ANDROMEDA-SHOCK. Inter-group differences (survivors vs. non-survivors at day-90). p=0.227; Time*Outcome. p=0.287

**Figure S4. Time-course of heart rate (HR) for survivors and non-survivors at day-90 in the preliminary cohort and ANDROMEDA-SHOCK**


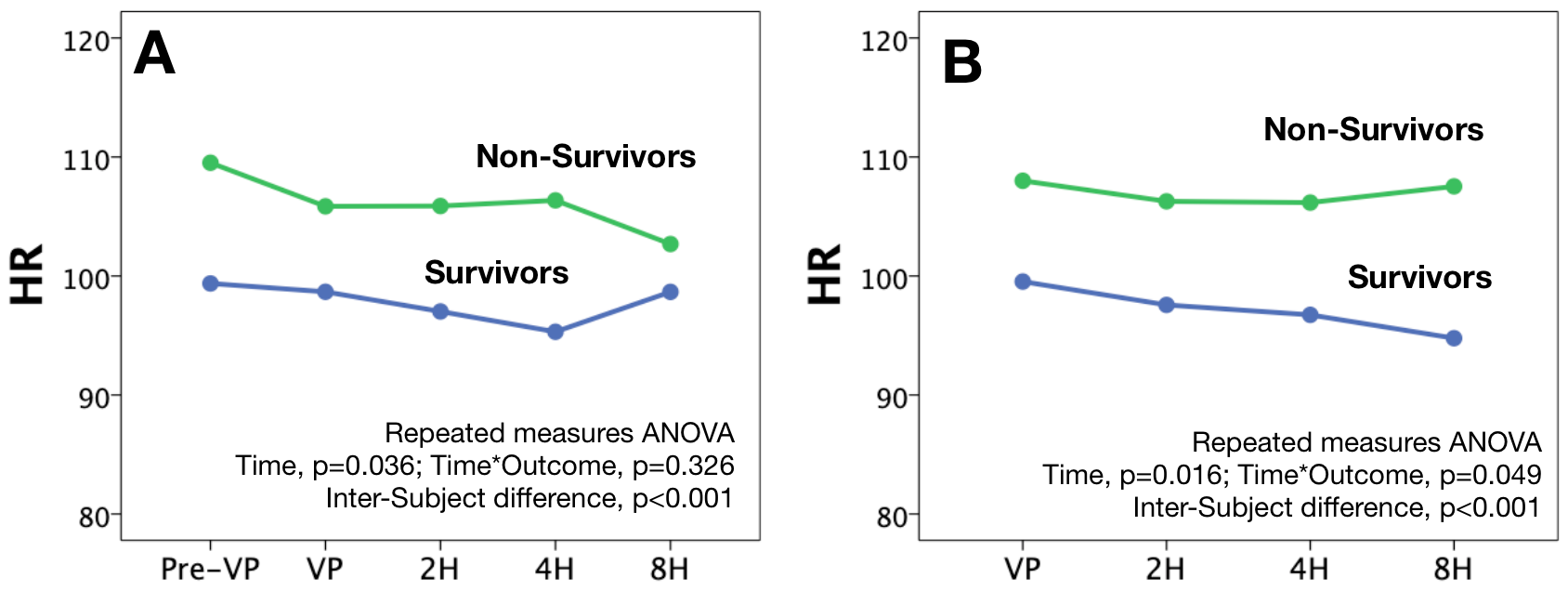


**Panel A.** Repeated Measures ANOVA, preliminary cohort. Inter-group differences (survivors vs. non-survivors at day-90). p<0.001; Time*Outcome. p=0.326

**Panel B.** Repeated Measures ANOVA, ANDROMEDA-SHOCK. Inter-group differences (survivors vs. non-survivors at day-90). p=0.227; Time*Outcome. p=0.049

**Figure S5. Time-course of mean arterial pressure (MAP) for survivors and non-survivors at day-90 in the preliminary cohort and ANDROMEDA-SHOCK**

**
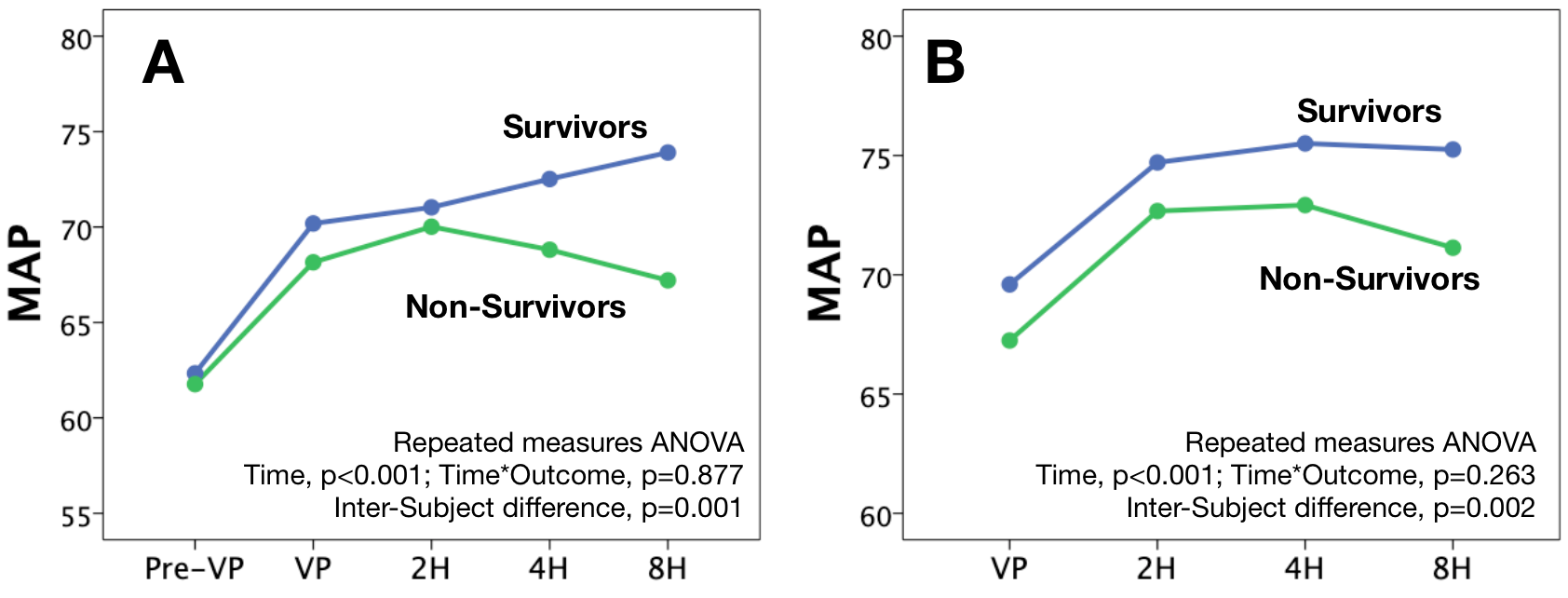
**

**Panel A.** Repeated Measures ANOVA, preliminary cohort. Inter-group differences (survivors vs. non-survivors at day-90). p=0.001; Time*Outcome. p=0.877

**Panel B.** Repeated Measures ANOVA, ANDROMEDA-SHOCK. Inter-group differences (survivors vs. non-survivors at day-90). p=0.002; Time*Outcome. p=0.263

**Figure S6. Time-course of pulse pressure (PP) for survivors and non-survivors at day-90 in the preliminary cohort and ANDROMEDA-SHOCK**


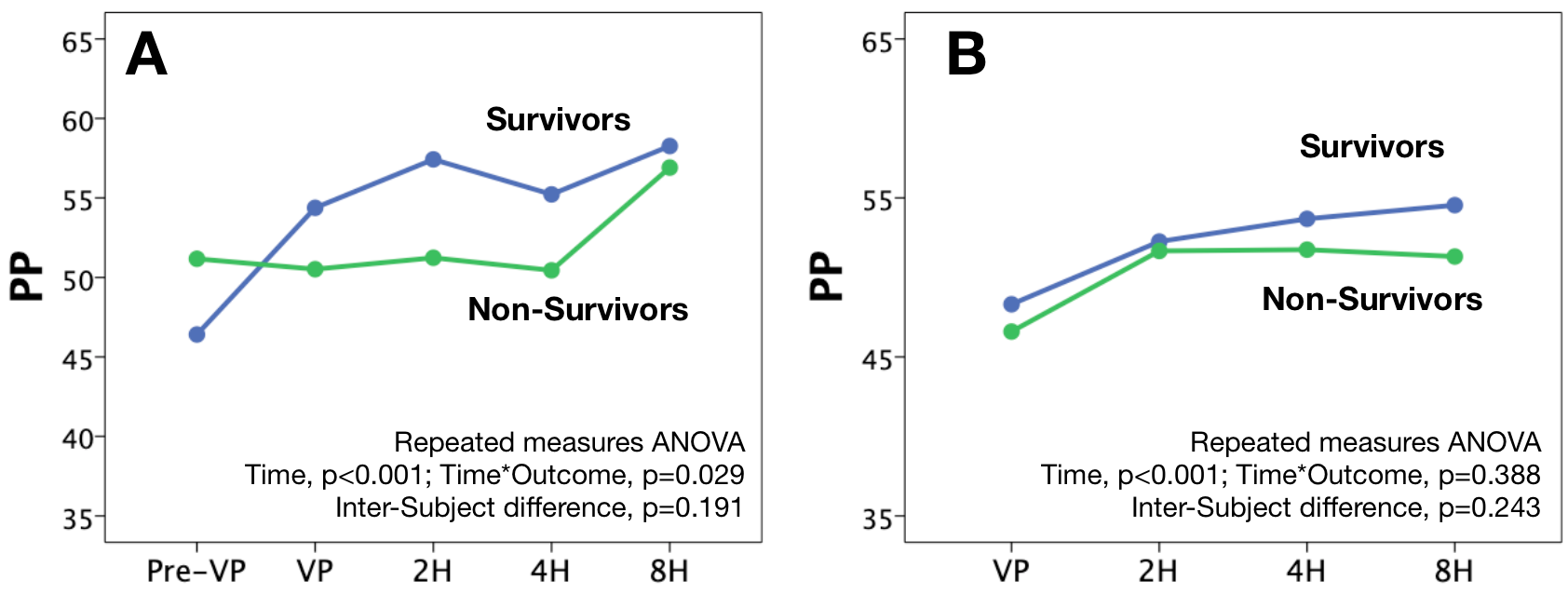


**Panel A.** Repeated Measures ANOVA, preliminary cohort. Inter-group differences (survivors vs. non-survivors at day-90). p=0.191; Time*Outcome. p=0.029

**Panel B.** Repeated Measures ANOVA, ANDROMEDA-SHOCK. Inter-group differences (survivors vs. non-survivors at day-90). p=0.243; Time*Outcome. p=0.388

**Figure S7. Time-course of systolic shock index for survivors and non-survivors at day-90 in the preliminary cohort and ANDROMEDA-SHOCK**


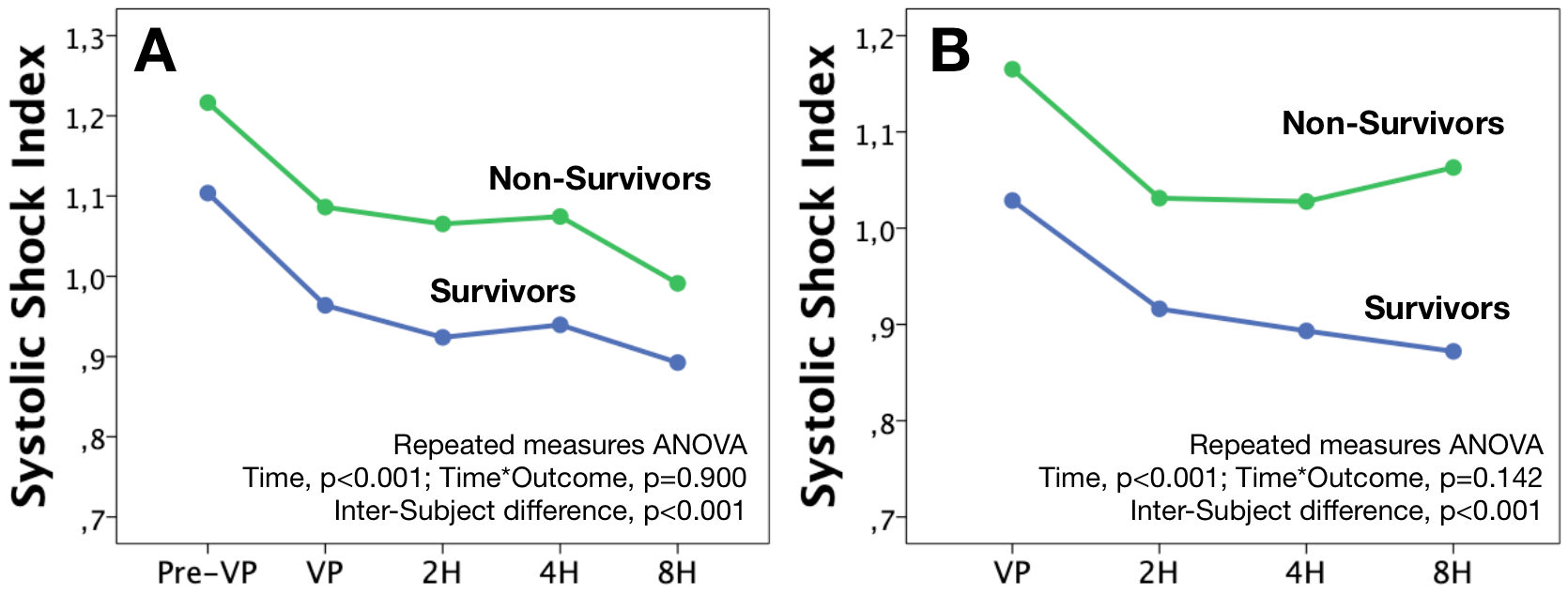


**Panel A.** Repeated Measures ANOVA, preliminary cohort. Inter-group differences (survivors vs. non-survivors at day-90). P<0.001; Time*Outcome. p=0.900

**Panel B.** Repeated Measures ANOVA, ANDROMEDA-SHOCK. Inter-group differences (survivors vs. non-survivors at day-90). P<0.001; Time*Outcome. p=0.142

**Figure S8a. Receiver operating characteristic (ROC) curves at Pre-VPs and 8-hours to predict mortality at day-28. Preliminary cohort**

**
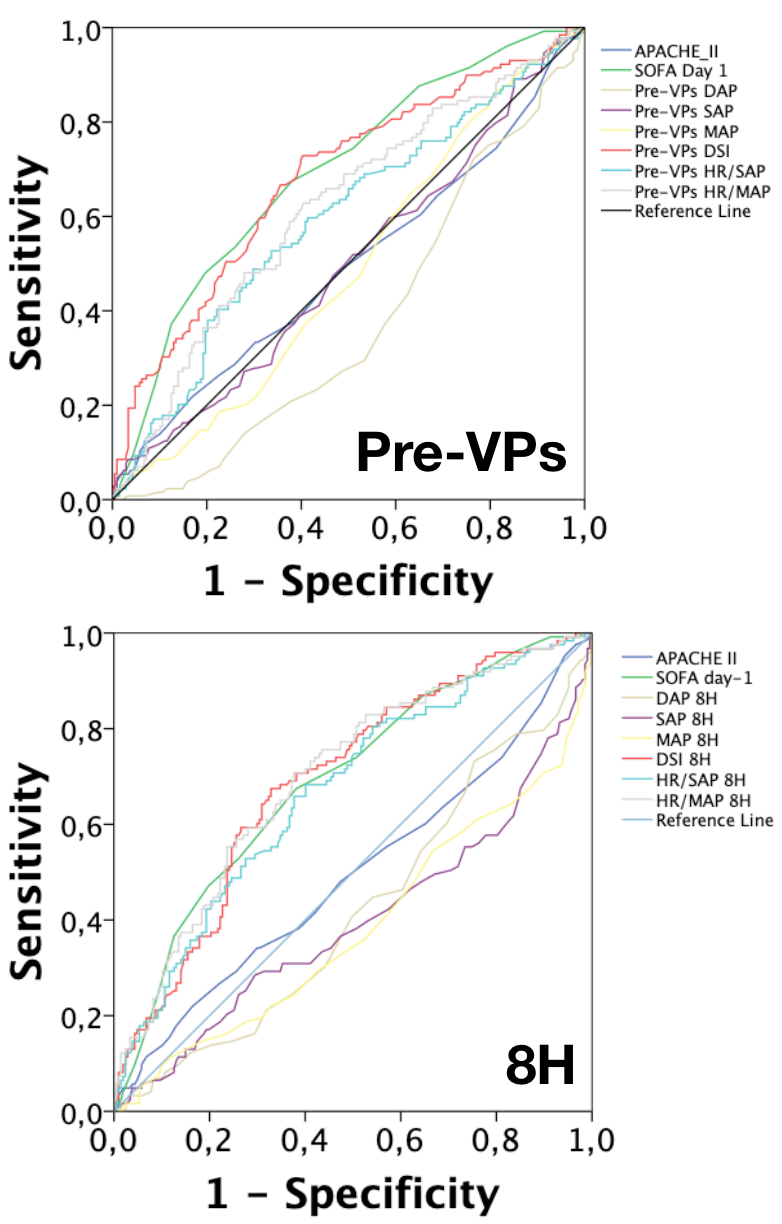
**

| **AUC – ROC Preliminary cohort (Pre-VPs): mortality day-28** | | | | | |
| --- | --- | --- | --- | --- | --- |
| **Variables** | **AUC** | **Error tip.** | **p** | **95% CI** | |
|  |  |  |  | **Lower Limit** | **Upper Limit** |
| APACHE_II | 0.495 | 0.033 | 0.865 | 0.429 | 0.56 |
| SOFA day-1 | 0.693 | 0.029 | 0.000 | 0.636 | 0.751 |
| Pre-VP DAP | 0.377 | 0.031 | 0.000 | 0.318 | 0.437 |
| Pre-VP SAP | 0.496 | 0.033 | 0.902 | 0.432 | 0.56 |
| Pre-VP MAP | 0.488 | 0.032 | 0.707 | 0.425 | 0.55 |
| Pre-VP DSI | 0.681 | 0.030 | 0.000 | 0.622 | 0.74 |
| Pre-VP HR/SAP ratio | 0.593 | 0.032 | 0.004 | 0.53 | 0.656 |
| Pre-VP HR/MAP ratio | 0.616 | 0.032 | 0.000 | 0.555 | 0.678 |

| **AUC – ROC Preliminary cohort (8-HOURS): mortality day-28** | | | | | |
| --- | --- | --- | --- | --- | --- |
| **Variables** | **AUC** | **Error tip.** | **p** | **95% CI** | |
|  |  |  |  | **Lower Limit** | **Upper Limit** |
| APACHE II | 0.495 | 0.034 | 0.888 | 0.429 | 0.562 |
| SOFA day-1 | 0.690 | 0.030 | 0.000 | 0.631 | 0.748 |
| DAP 8H | 0.420 | 0.032 | 0.015 | 0.357 | 0.484 |
| SAP 8H | 0.398 | 0.034 | 0.002 | 0.332 | 0.464 |
| MAP 8H | 0.383 | 0.033 | 0.000 | 0.319 | 0.448 |
| DSI 8H | 0.692 | 0.030 | 0.000 | 0.634 | 0.75 |
| HR/SAP 8H | 0.669 | 0.031 | 0.000 | 0.609 | 0.729 |
| HR/MAP 8H | 0.700 | 0.030 | 0.000 | 0.642 | 0.758 |

**Figure S8b. Receiver operating characteristic (ROC) curves at Pre-VPs and 8-hours to predict mortality at day-90. Preliminary cohort**


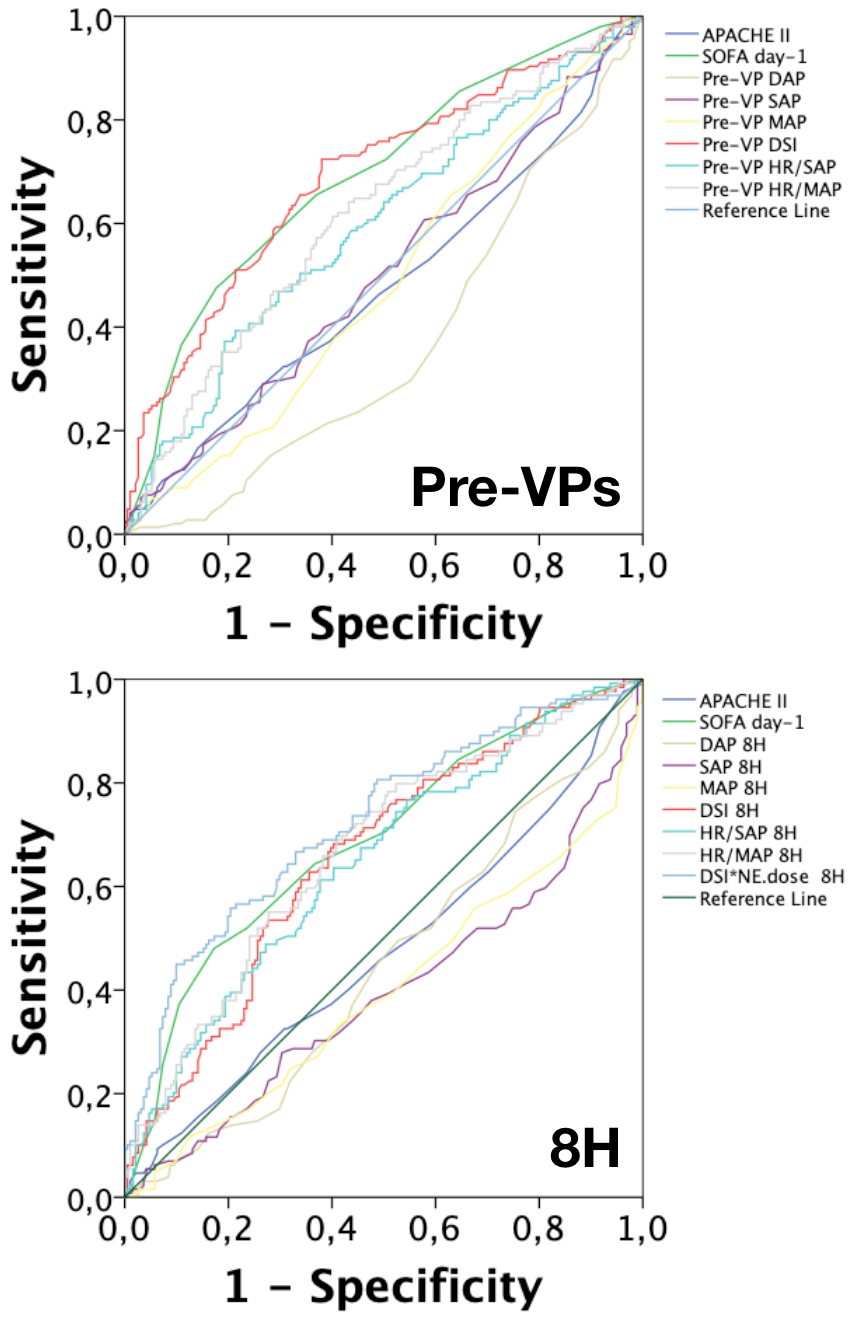


| **AUC – ROC Preliminary cohort (Pre-VPs): mortality day-90** | | | | | |
| --- | --- | --- | --- | --- | --- |
| **Variables** | **AUC** | **Error tip.** | **p** | **95% CI** | |
|  |  |  |  | **Lower Limit** | **Upper Limit** |
| APACHE_II | 0.479 | 0.032 | 0.499 | 0.415 | 0.542 |
| SOFA day-1 | 0.691 | 0.029 | 0.000 | 0.634 | 0.748 |
| Pre-VP DAP | 0.367 | 0.030 | 0.000 | 0.308 | 0.426 |
| Pre-VP SAP | 0.502 | 0.032 | 0.941 | 0.44 | 0.565 |
| Pre-VP MAP | 0.487 | 0.032 | 0.690 | 0.425 | 0.549 |
| Pre-VP DSI | 0.690 | 0.029 | 0.000 | 0.633 | 0.748 |
| Pre-VP HR/SAP ratio | 0.595 | 0.031 | 0.003 | 0.533 | 0.656 |
| Pre-VP HR/MAP ratio | 0.620 | 0.031 | 0.000 | 0.559 | 0.68 |

| **AUC – ROC Preliminary cohort (8-HOURS): mortality day-90** | | | | | |
| --- | --- | --- | --- | --- | --- |
| **Variables** | **AUC** | **Error tip.** | **p** | **95% CI** | |
|  |  |  |  | **Lower Limit** | **Upper Limit** |
| APACHE II | 0.475 | 0.033 | 0.441 | 0.409 | 0.540 |
| SOFA day-1 | 0.687 | 0.030 | 0.000 | 0.627 | 0.747 |
| DAP 8H | 0.447 | 0.033 | 0.105 | 0.383 | 0.510 |
| SAP 8H | 0.396 | 0.033 | 0.002 | 0.331 | 0.461 |
| MAP 8H | 0.398 | 0.033 | 0.002 | 0.333 | 0.462 |
| DSI 8H | 0.657 | 0.031 | 0.000 | 0.596 | 0.717 |
| HR/SAP 8H | 0.646 | 0.031 | 0.000 | 0.585 | 0.707 |
| HR/MAP 8H | 0.668 | 0.031 | 0.000 | 0.608 | 0.729 |
| DSI * NE.dose 8H | 0.723 | 0.029 | 0.000 | 0.665 | 0.780 |

**Figure S9a. Receiver operating characteristic (ROC) curves at VPs and 8-hours to predict mortality at day-28. ANDROMEDA-SHOCK**

**
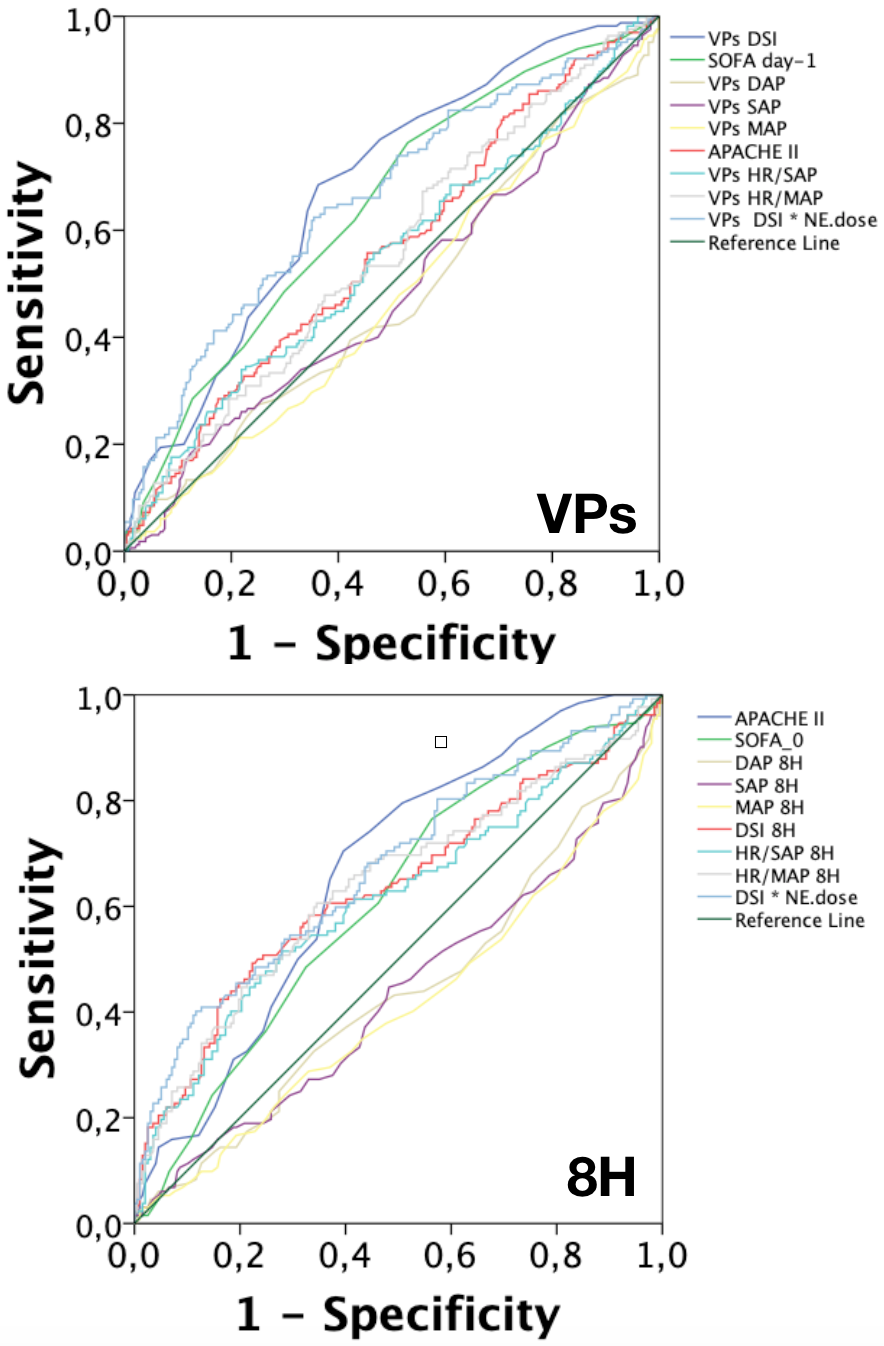
**

| **AUC – ROC ANDROMEDA SHOCK (VPs): mortality day-28** | | | | | |
| --- | --- | --- | --- | --- | --- |
| **Variables** | **AUC** | **Error tip.** | **p** | **95% CI** | |
|  |  |  |  | **Lower Limit** | **Upper Limit** |
| APACHE_II | 0.563 | 0.029 | 0.031 | 0.506 | 0.619 |
| SOFA day-1 | 0.643 | 0.027 | 0.000 | 0.59 | 0.697 |
| VP DAP | 0.473 | 0.029 | 0.358 | 0.416 | 0.531 |
| VP SAP | 0.485 | 0.029 | 0.612 | 0.428 | 0.543 |
| VP MAP | 0.470 | 0.029 | 0.302 | 0.413 | 0.527 |
| VP DSI | 0.680 | 0.026 | 0.000 | 0.629 | 0.732 |
| VP HR/SAP ratio | 0.547 | 0.029 | 0.107 | 0.489 | 0.604 |
| VP HR/MAP ratio | 0.560 | 0.029 | 0.039 | 0.503 | 0.616 |
| VP DSI * NE.dose | 0.659 | 0.028 | 0.000 | 0.605 | 0.713 |

| **AUC – ROC Preliminary cohort (8H): mortality day-28** | | | | | |
| --- | --- | --- | --- | --- | --- |
| **Variables** | **AUC** | **Error tip.** | **p** | **95% CI** | |
|  |  |  |  | **Lower Limit** | **Upper Limit** |
| APACHE II | 0.665 | 0.030 | 0.000 | 0.607 | 0.724 |
| SOFA day-1 | 0.611 | 0.031 | 0.001 | 0.55 | 0.672 |
| DAP 8H | 0.439 | 0.033 | 0.059 | 0.374 | 0.503 |
| SAP 8H | 0.435 | 0.033 | 0.046 | 0.371 | 0.5 |
| MAP 8H | 0.413 | 0.033 | 0.007 | 0.349 | 0.477 |
| DSI 8H | 0.634 | 0.032 | 0.000 | 0.571 | 0.698 |
| HR/SAP 8H | 0.615 | 0.033 | 0.000 | 0.551 | 0.679 |
| HR/MAP 8H | 0.638 | 0.032 | 0.000 | 0.575 | 0.701 |
| DSI * NE.dose 8H | 0.674 | 0.031 | 0.000 | 0.614 | 0.734 |

**Figure S9b. Receiver operating characteristic (ROC) curves at VPs and 8-hours to predict mortality at day-90. ANDROMEDA-SHOCK**

**
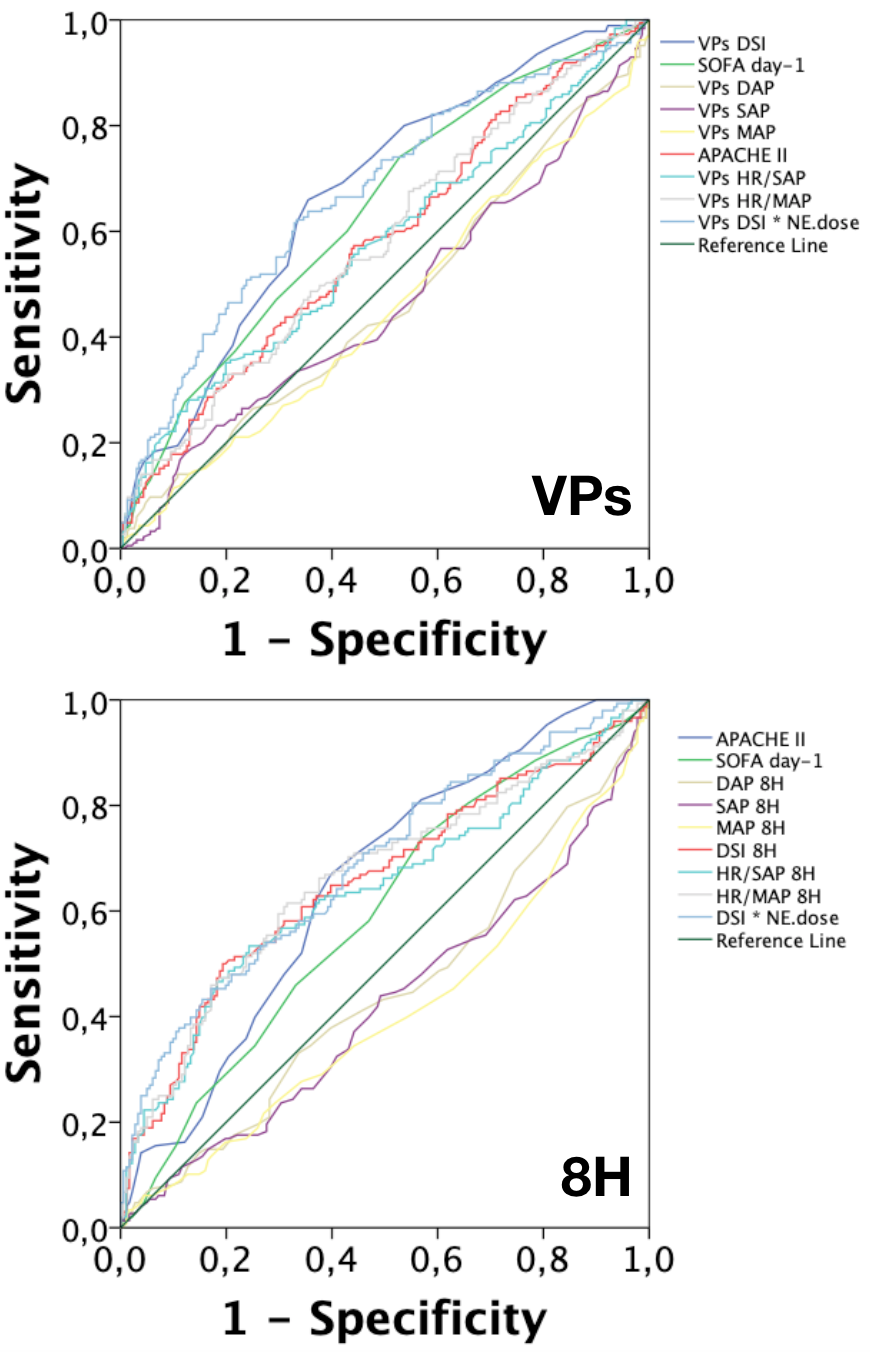
**

| **AUC – ROC ANDROMEDA SHOCK (VPs): mortality day-90** | | | | | |
| --- | --- | --- | --- | --- | --- |
| **Variables** | **AUC** | **Error tip.** | **p** | **95% CI** | |
|  |  |  |  | **Lower Limit** | **Upper Limit** |
| APACHE_II | 0.580 | 0.028 | 0.005 | 0.525 | 0.635 |
| SOFA day-1 | 0.636 | 0.027 | 0.000 | 0.583 | 0.689 |
| VP DAP | 0.467 | 0.029 | 0.244 | 0.410 | 0.523 |
| VP SAP | 0.467 | 0.029 | 0.240 | 0.410 | 0.523 |
| VP MAP | 0.455 | 0.029 | 0.116 | 0.399 | 0.511 |
| VP DSI | 0.671 | 0.026 | 0.000 | 0.620 | 0.723 |
| VP HR/SAP ratio | 0.574 | 0.029 | 0.010 | 0.518 | 0.630 |
| VP HR/MAP ratio | 0.582 | 0.028 | 0.004 | 0.527 | 0.637 |
| VP DSI * NE.dose | 0.670 | 0.027 | 0.000 | 0.618 | 0.723 |

| **AUC – ROC ANDROMEDA SHOCK (8H): mortality day-90** | | | | | |
| --- | --- | --- | --- | --- | --- |
| **Variables** | **AUC** | **Error tip.** | **p** | **95% CI** | |
|  |  |  |  | **Lower Limit** | **Upper Limit** |
| APACHE II | 0.651 | 0.030 | 0.000 | 0.592 | 0.71 |
| SOFA day-1 | 0.593 | 0.031 | 0.004 | 0.531 | 0.654 |
| DAP 8H | 0.446 | 0.032 | 0.095 | 0.384 | 0.509 |
| SAP 8H | 0.423 | 0.032 | 0.016 | 0.36 | 0.485 |
| MAP 8H | 0.406 | 0.032 | 0.003 | 0.344 | 0.468 |
| DSI 8H | 0.655 | 0.031 | 0.000 | 0.595 | 0.716 |
| HR/SAP 8H | 0.641 | 0.031 | 0.000 | 0.580 | 0.702 |
| HR/MAP 8H | 0.662 | 0.031 | 0.000 | 0.602 | 0.722 |
| DSI * NE.dose 8H | 0.686 | 0.029 | 0.000 | 0.629 | 0.744 |

**Table S6. Early start vasopressor (VE-VPs) and mortality by quintiles of Pre-VPs/DSI. Preliminary Cohort**

| **Quintile** | **Vasopressor** | **Survivor** | **Non-Survivor** | **Total** | **Chi-square** | **p** |
| --- | --- | --- | --- | --- | --- | --- |
| **1** | <1H (VE-VPs) | 18 (90.0) | 2 (10.0) | 20 | 3.400 | 0.075 |
|  | >1H (D-VPs) | 33 (68.8) | 15 (31.2) | 48 |  |  |
| **2** | <1H (VE-VPs) | 21 (84.0) | 4 (16.0) | 25 | 2.998 | 0.100 |
|  | >1H (D-VPs) | 27 (64.3) | 15 (37.7) | 42 |  |  |
| **3** | <1H (VE-VPs) | 14 (77.8) | 4 (22.2) | 18 | 3.575 | 0.091 |
|  | >1H (D-VPs) | 25 (52.1) | 23 (47.9) | 48 |  |  |
| **4** | <1H (VE-VPs) | 8 (57.1) | 6 (42.9) | 14 | 0.611 | 0.55 |
|  | >1H (D-VPs) | 25 (45.5) | 30 (54.5) | 55 |  |  |
| **5** | <1H (VE-VPs) | 9 (56.2) | 7 (43.8) | 16 | 6.059 | 0.028 |
|  | >1H (D-VPs) | 12 (23.5) | 39 (76.5) | 51 |  |  |

**VE-VPs: Very early start of vasopressor support.**

A very early start of vasopressor was defined as the one started within the first hour of receiving the first fluid load with resuscitative intention**.**

**D-VPs: Delayed start of vasopressor support.**

A delayed start of vasopressor was defined as the one started >1 hour of receiving the first fluid load with resuscitative intention.

**Table S7. Multivariate logistic regression for the effect of very early start of vasopressor on mortality at 90-day at each Pre-VPs/DSI quintile. Preliminary Cohort**

| Variable | β Coefficient | Exp (β) | 95%CI | p |
| --- | --- | --- | --- | --- |
| **Quintile 1**  VE-VPs | 1.531 | 4.623 | 0.829 – 25.773 | 0.081 |
| SOFA | 0.189 | 1.208 | 0.987 – 1.477 | 0.416 |
| Lactate | 0.213 | 1.237 | 0.999 – 1.533 | 0.051 |
| **Quintile 2**  VE-VPs | 0.952 | 2.591 | 0.634 – 10.597 | 0.185 |
| SOFA | 0.387 | 1.473 | 1.156 – 1.878 | 0.002 |
| Lactate | 0.102 | 1.108 | 0.867 – 1.415 | 0.412 |
| **Quintile 3**  VE-VPs | 1.382 | 3.983 | 1.000 – 15.683 | 0.051 |
| SOFA | 0.193 | 1.213 | 1.034 – 1.422 | 0.018 |
| Lactate | 0.138 | 1.148 | 0.926 – 1.423 | 0.208 |
| **Quintile 4**  VE-VPs | 0.201 | 1.222 | 0.335 – 4.465 | 0.762 |
| SOFA | 0.070 | 1.072 | 0.900 – 1,277 | 0.437 |
| Lactate | 0.304 | 1.356 | 1.078 – 1.705 | 0.009 |
| **Quintile 5**  VE-VPs | 1.838 | 6.285 | 1.581 – 24.979 | 0.009 |
| SOFA | 0.218 | 1.243 | 1.036 – 1.491 | 0.019 |
| Lactate | 0.112 | 1.119 | 0.908 – 1.377 | 0.291 |

**VE-VPs: Very early start of vasopressor support.**

A very early start of vasopressor was defined as the one started within the first hour of receiving the first fluid load with resuscitative intention**.**

**Table S8. General characteristics, hemodynamics, perfusion parameters, fluids, vasopressors and clinical outcomes. Preliminary cohort**

|  | **All** | **VE-VPs**  **(n=93)** | **D-VPs**  **(n=244)** | **p** |
| --- | --- | --- | --- | --- |
| **General characteristics** |  |  |  |  |
| Age, years | 64 (51 – 74) | 63 (51 – 74) | 64 (51 – 74) | 0.99 |
| Male sex, n (%) |  |  |  |  |
| Weight, Kg | 68 (59 – 76) | 70 (57 – 80) | 67 (59 – 75) | 0.11 |
| APACHE II | 16 (13 – 22) | 16 (13 – 19) | 17 (13 – 23) | 0.07 |
| SOFA day-1 | 9 (7 – 12) | 7 (8 – 12) | 9 (7 – 12) | 0.90 |
| Infection Source, n (%) |  |  |  |  |
| Lung | 109 (32.4) | 33 (35.9) | 76 (31.1) | 0.43 |
| Genitourinary | 64 (19.0) | 17 (18.3) | 47 (19.3) | 0.88 |
| Abdominal | 114 (33.8) | 29 (31.2) | 85 (34.8) | 0.61 |
| Soft tissue | 31 (9.2) | 9 (9.7) | 22 (9.0) | 0.84 |
| Bacteremia | 80 (23.7) | 17 (18.3) | 63 (25.8) | 0.16 |
| Other | 16 (4.7) | 7 (7.5) | 9 (3.7) | 0.16 |
| Origin |  |  |  | 0.62 |
| Emergency room | 226 (67.1) | 66 (71.0) | 160 (66.0) |  |
| General ward | 48 (14.2) | 11 (11.8) | 37 (15.2) |  |
| Intensive care unit | 63 (18.7) | 16 (17) | 47 (19.3) |  |
| Comorbidities, n (%) |  |  |  |  |
| Hypertension | 124 (36.9) | 34 (36.6) | 90 (37.0) | 1.0 |
| Chronic Coronary disease | 17 (5.0) | 4 (4.3) | 13 (5.3) | 1.0 |
| Chronic Heart Failure | 30 (8.9) | 7 (7.5) | 23 (9.5) | 0.67 |
| ESRF | 21 (6.2) | 5 (5.4) | 16 (6.6) | 0.80 |
| Previous Stroke | 8 (2.4) | 3 (3.2) | 5 (2.0) | 0.69 |
| Chronic Atrial Fibrillation | 19 (5.6) | 6 (6.5) | 13 (5.3) | 0.79 |
| Diabetes | 68 (20.2) | 18 (19.4) | 50 (20.6) | 0.88 |
| Cancer | 67 (19.9) | 21 (22.6) | 46 (18.9) | 0.45 |
| COPD | 36 (10.7) | 14 (15.1) | 22 (9.1) | 0.12 |
| Autoimmune disease | 17 (5.1) | 1 (1.1) | 16 (6.6) | 0.05 |
| Chronic use steroids | 45 (13.4) | 9 (9.7) | 36 (14.8) | 0.28 |
| Cirrhosis | 26 (7.7) | 4 (4.3) | 22 (9.1) | 0.16 |
| Acute Myocardial Infarction | 6 (1.8) | 22 (2.2) | 4 (1.6) | 0.67 |
| Acute Heart Failure | 20 (5.9) | 8 (8.6) | 12 (4.9) | 0.20 |
| Acute Stroke | 11 (3.3) | 2 (2.2) | 9 (3.7) | 0.73 |
| Acute Atrial Fibrillation | 13 (3.9) | 2 (2.2) | 11 (4.5) | 0.53 |

APACHE II: Acute physiology and chronic health evaluation; SOFA: Sequential organ failure assessment; ESRF: end-stage renal failure; COPD: chronic obstructive pulmonary disease.

VE-VPs: very early start of vasopressor (vasopressor started <1-hour from the first fluid load with resuscitative intention)

D-VPs: delayed start of vasopressor (vasopressor started >1-hour from the first fluid load with resuscitative intention)
